# The cancer-associated CTCFL/BORIS protein targets multiple classes of genomic repeats, with a distinct binding and functional preference for humanoid-specific SVA transposable elements

**DOI:** 10.1186/s13072-016-0084-2

**Published:** 2016-08-31

**Authors:** Elena M. Pugacheva, Evgeny Teplyakov, Qiongfang Wu, Jingjing Li, Cheng Chen, Chengcheng Meng, Jian Liu, Susan Robinson, Dmitry Loukinov, Abdelhalim Boukaba, Andrew Paul Hutchins, Victor Lobanenkov, Alexander Strunnikov

**Affiliations:** 1 Molecular Epigenetics Laboratory, Guangzhou Institutes of Biomedicine and Health, Guangzhou, 510530 Guangdong China; 2 Laboratory of Immunogenetics, NIH, NIAID, Rockville, MD 20852 USA; 3 Department of Biology, Southern University of Science and Technology of China, Shenzhen, 518055 Guangdong China

## Abstract

**Background:**

A common aberration in cancer is the activation of germline-specific proteins. The DNA-binding proteins among them could generate novel chromatin states, not found in normal cells. The germline-specific transcription factor BORIS/CTCFL, a paralog of chromatin architecture protein CTCF, is often erroneously activated in cancers and rewires the epigenome for the germline-like transcription program. Another common feature of malignancies is the changed expression and epigenetic states of genomic repeats, which could alter the transcription of neighboring genes and cause somatic mutations upon transposition. The role of BORIS in transposable elements and other repeats has never been assessed.

**Results:**

The investigation of BORIS and CTCF binding to DNA repeats in the K562 cancer cells dependent on BORIS for self-renewal by ChIP-chip and ChIP-seq revealed three classes of occupancy by these proteins: elements cohabited by BORIS and CTCF, CTCF-only bound, or BORIS-only bound. The CTCF-only enrichment is characteristic for evolutionary old and inactive repeat classes, while BORIS and CTCF co-binding predominately occurs at uncharacterized tandem repeats. These repeats form staggered cluster binding sites, which are a prerequisite for CTCF and BORIS co-binding. At the same time, BORIS preferentially occupies a specific subset of the evolutionary young, transcribed, and mobile genomic repeat family, SVA. Unlike CTCF, BORIS prominently binds to the VNTR region of the SVA repeats in vivo. This suggests a role of BORIS in SVA expression regulation. RNA-seq analysis indicates that BORIS largely serves as a repressor of SVA expression, alongside DNA and histone methylation, with the exception of promoter capture by SVA.

**Conclusions:**

Thus, BORIS directly binds to, and regulates SVA repeats, which are essentially movable CpG islands, via clusters of BORIS binding sites. This finding uncovers a new function of the global germline-specific transcriptional regulator BORIS in regulating and repressing the newest class of transposable elements that are actively transposed in human genome when activated. This function of BORIS in cancer cells is likely a reflection of its roles in the germline.

**Electronic supplementary material:**

The online version of this article (doi:10.1186/s13072-016-0084-2) contains supplementary material, which is available to authorized users.

## Background

Transposable elements (TEs) play active roles in normal genome evolution in humans [1] and in primates in general [2], as well as in sporadic genome rearrangement [3–5] including deleterious events associated with pathology [6–12]. Multiple polymorphisms and intron evolution in normal human populations are largely facilitated by TE insertions [13, 14]. A substantial and distinct role of satellite repeats was also recently demonstrated for double-strand breaks (DSBs) incidence upon replication stress [15]. Active families of TEs (L1, Alu, and SVA) account for a large number of germline mutations [16]. In cancer, insertions of mobile element and the recombination between them have been identified as causes of many cancers [12, 17, 18], with some repeats shown to become aberrantly expressed [17, 19] to acquire a potential to change the regulation of neighboring genes [17, 20, 21] and to destabilize chromosomes [7, 22]. The effect of repeated DNA in the origins and progression of cancer and tumor cell physiology could be two-pronged: the induced change of expression in neighboring or targeted genes [22–24] and the structural destabilization of the epigenetic landscape of chromosomes [2, 25]. These two effects are interrelated, as epigenetic changes in the repeats open chromosomal domains for both aberrant changes in gene expression and elevated somatic recombination. Some elements were also shown to act as bona fide enhancers [26].

The presence of a strong epigenetic component in such repeats and TE-mediated genome regulation and instability is well established [20, 27–30]. In cancer cells, there is likely a higher epigenetic impact of TEs, compared to the norm [12], as promoters of expressed mobile elements become hypomethylated and their transcription elevated [22, 31, 32].

The array of epigenetic changes leading to repeat deregulation in cancer cannot be understood without molecular analysis of repeats’ chromatin. This brings to light the role of CTCF and its paralog CTCFL/BORIS in these processes. In addition to serving as a bona fide transcription factor, CTCF reads the epigenetic marks [33–36] and plays a key role in the formation of topologically associated domains (TADs) in chromatin [37–39], in remodeling chromatin structure [40], and in the formation of chromatin boundaries [29, 41]. CTCF was also shown to have multiple binding sites embedded in TEs [42, 43]. CTCF target sites (CTSs) are also important for telomere repeat stability [44, 45]. Furthermore, the fact that CTCF control of gene expression and recombination requires physical contacts between different CTSs via looping [46–49] indicates that CTCF sites in repeats are not inert in the chromatin architecture, as indeed was demonstrated at some instances [50–53].

Taking into account the important role of CTCF in regulating TE expression and epigenetic maintenance, it is possible that the aberrant activation of its germline paralog CTCFL/BORIS in cancer has an impact on repeat physiology and genome stability. BORIS is a cancer testis (CT) gene [54], and its ectopic expression could be lethal or inhibitory for somatic cells because BORIS, being a germline transcription factor, activates gene expression of germline-specific genes on its own or in cooperation with CTCF [55]. Nevertheless, some cancer cells undergo adaptation/addiction to BORIS activation and incorporate the BORIS protein into their physiology [55, 56]. BORIS also interferes with a variety of other CTCF-specific functions in somatic cells, such as in the organization of chromatin loops that are alternative to the chromatin configuration of normal cells [55]. The ultimate molecular and physiological role of BORIS in cancer is still poorly understood, however, beyond the association with stemness [56], phenocopying of germline-specific gene expression pattern, and the corresponding 3D chromatin organization [55]. In particular, it is not clear how some cancer cells became dependent on BORIS for their proliferation, making BORIS a potential anticancer target [57, 58].

While many genomic repeats are heavily methylated and BORIS has a probable role in DNA demethylation [57, 59–61], the role of BORIS in repeat biology has not been studied. Incidentally, even the most comprehensive genome-wide studies on CTCF tended to ignore the possible simultaneous presence of BORIS in cells studied, be it cancer or embryonic stem cells [48, 50, 62–64]. In this present study, we attempted to assess the specific pattern of BORIS recognition of genomic repeats in cancer cells and to link it to TE expression. As a result, we uncovered a surprising association of BORIS with one of the evolutionary youngest families of actively transcribed and mobile repeats in human genome, the SVA family of TEs. Follow-up analysis of the modulation of BORIS expression revealed that it predominately acts as one of the mechanisms repressing the expression of these elements.

## Results

### BORIS expression in K562 forms a specific pattern of repeat binding

**Fig. 1:**
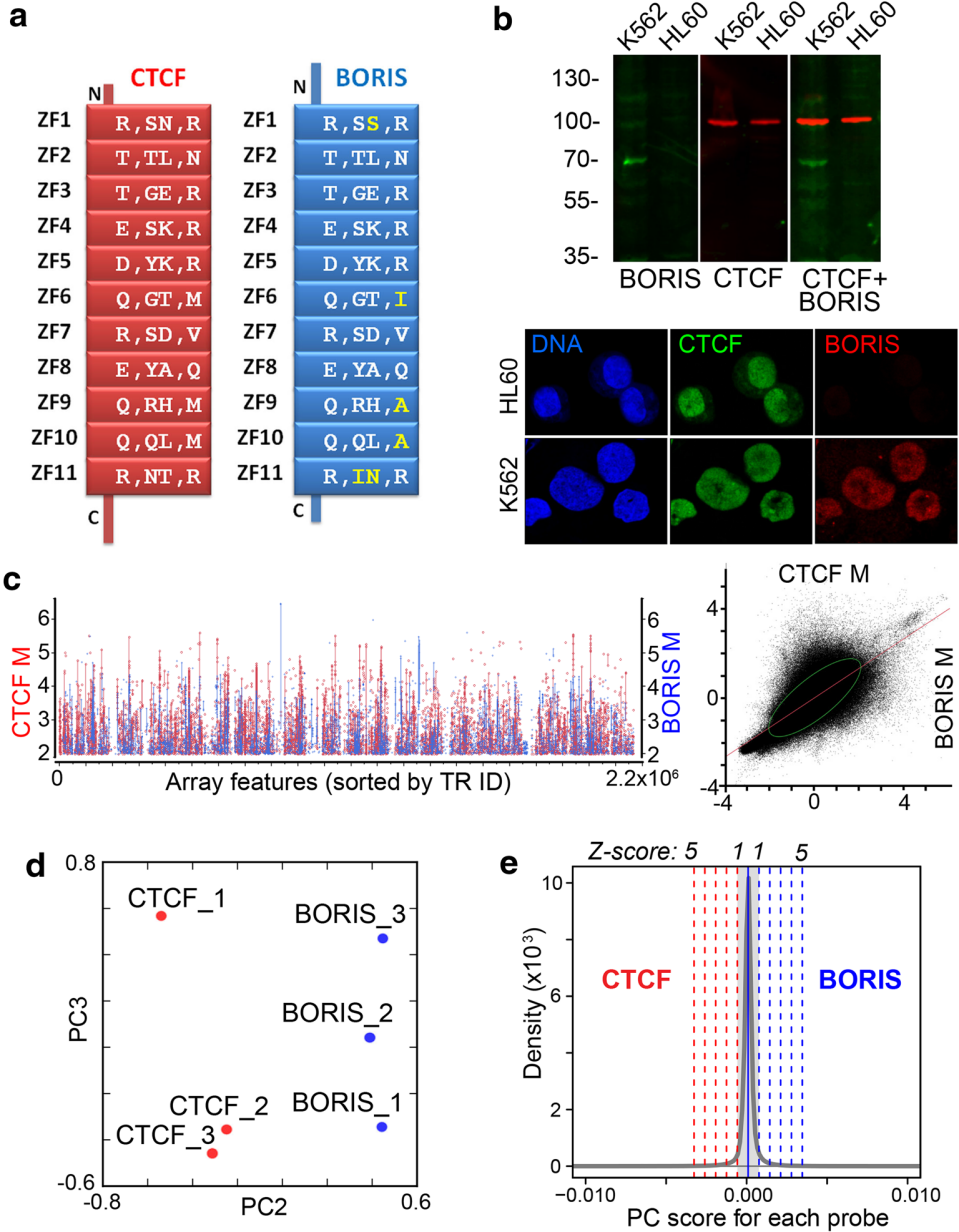
BORIS expression in K562 establishes a definitive pattern of repeat binding. **a** A schematic of CTCF and BORIS proteins with the four amino acids residues essential for DNA recognition by each zinc finger (ZFs) showing. The minor differences, indicated in *yellow*, do not affect the DNA-binding specificity in vitro neither the consensus derived from the genome-wide binding study [55]. **b** LI-COR image of immunoblotting for BORIS and CTCF proteins in whole-cell protein extracts of K562 (BORIS positive) and HL60 (BORIS negative) cancer cell lines of myeloid origin. *Below* the immunofluorescent and DNA staining of the two corresponding cell lines. **c** The *left panel* shows the enrichment ratio (M) for CTCF and BORIS across all the tiles of the TR microarray. *Dots* represent microarray tiles enriched ≥4 by either CTCF (*red*) or BORIS (*blue*) with *lines* connecting different tiles belonging to the same repeat. SAM showed that 42,715 tiles were differentially occupied with FDR ranging from 0.103 to 0.245, with 0.75 correlations between CTCF and BORIS arrays. Both measures indicate that a minority of the repeats were differentially bound by the two proteins. *The right panel* shows the linear fit of BORIS M ratios by CTCF M ratios, with the fit *line* and 95 % bivariate normal *ellipse* displayed. **d** Principal component analysis of ChIP-chip data from K562. PCA was performed with singular value decomposition (SVD), and the first principal component describing the trend of the data was excluded from the analysis. PC2 (42 %) explains the difference between CTCF and BORIS experiments, and PC3 (16 %) explains variance between the replicates. **e** Smoothed histogram for probe loading kernel density (*Y* axis) estimate for all probes along the PC2 axis. The mean is indicated, as well as the number of standard deviations from the mean (*Z*-score). Most probes show no significant loading on PC2, and no significant difference between CTCF and BORIS. Only a fraction of probes show a significant contribution to the PC2 axis

We have previously shown that tandem repeats (TRs) in a human cancer cell line may serve as foci for multiple DNA damage events induced upon the resolution of mitotic chromosome bridges [65]. In that study, custom repeat microarray ChIP-chip was used to validate some of the enrichments identified in the preceding ChIP-seq analysis. The need for a two-method validation procedure stems from the fact that at present there is no unbiased way to align short next-generation sequencing (NGS) reads to massively repeated DNA, while microarray analysis has well documented limitations of its own. Here, we employed a similar two-step approach in reverse; the repeats’ enrichment by DNA-binding proteins was first assessed by ChIP-chip and then validated by ChIP-seq. We used mainly the established cancer cell line K562 as a model for the coexistence of CTCF and BORIS stably expressed at a relatively the same level, as assayed by RT-PCR [55], to assess genome repeat occupancy by these two proteins. K562 retains a set of properties characteristic for cancer stem cells, e.g., the ability to initiate tumors in graft models, and the propensity to differentiate in response to exogenous stimuli [66]. As CTCF and BORIS have essentially the same composition of the DNA-binding domain, including the number of ZF and their spacing, as well as residues involved in DNA contacts (Fig. 1a), they show the virtually identical DNA-binding specificity in vitro, albeit not in native chromatin [55]. Therefore, it was important to use a cell line where two proteins are expressed in equivalent amounts, such as K562. Unlike most established cancer cell lines or primary non-germline tumor cells, where the expression of BORIS is low, with only a minor subset of cells characterized by high BORIS expression [56], K562 expresses high level of BORIS largely localized to the nuclei (Fig. 1b). BORIS was also confirmed to be incorporated into transcription regulation in K562 and to be required for its self-renewal [55].

For the initial analysis, by ChIP-chip, anti-CTCF and anti-BORIS immunoprecipitations were conducted and microarray hybridization was performed as described in Methods. The plot of normalized ChIP-chip fluorescence intensities showed indications of distinct binding patterns for BORIS and CTCF on highly enriched tiles (Fig. 1c). Significance analysis of microarray (SAM) indicated that over 40,000 tiles were enriched differentially by CTCF and BORIS, but provided little clue about the occupancy of the rest of the repeats. The principal component analysis (PCA) of arrays hybridized to CTCF and BORIS ChIP samples confirmed the presence of differentially bound genomic repeats (Fig. 1d). The PCA also revealed the three expected scenarios of occupancy: binding by BORIS only, by CTCF only, and BORIS and CTCF co-binding being by far the largest group (Fig. 1e). As CTCF and BORIS have essentially the same DNA-binding specificities in vitro, the differences in occupancy observed in vivo must be largely driven by the epigenetic factors.

Prior to proceeding further with analyses of repeat binding sequences, we conducted a validation of ChIP-chip data using an alternative high-throughput procedure, ChIP-seq, as conventional qPCR validation methods are not applicable or scalable to the TRs genome-wide. We set out to validate the three identified subsets: first, repeats preferentially enriched by CTCF (Fig. 1e), second, repeats preferentially enriched by BORIS (Fig. 1e), and, third, repeats equally enriched by both CTCF and BORIS (Fig. 1e, a subset of the middle group). Based on detailed PCA analysis, an additional cutoff across the three groups was applied to make uniform criteria for selecting the representative subsets for validation. For co-bound repeats we chose the 4× enrichment for both proteins in all three ChIP-chip replicates, while for the Z5 groups we used 4× enrichment for one protein, with no enrichment for the other, also in all three replicates. Drawing the threshold at such a relatively high level also significantly reduced repeat redundancy in the TR dataset. For the ChIP-seq validation, we considered a ChIP-chip-positive repeat validated, if any tile from that repeat was reproducibly enriched at least twofold in ChIP-seq datasets with 95 % DNA match. Thus, all the repeats discussed below are repeats identified by ChIP-chip and validated by ChIP-seq.

### Co-binding of BORIS and CTCF is characteristic for the simple tandem repeats

**Fig. 2:**
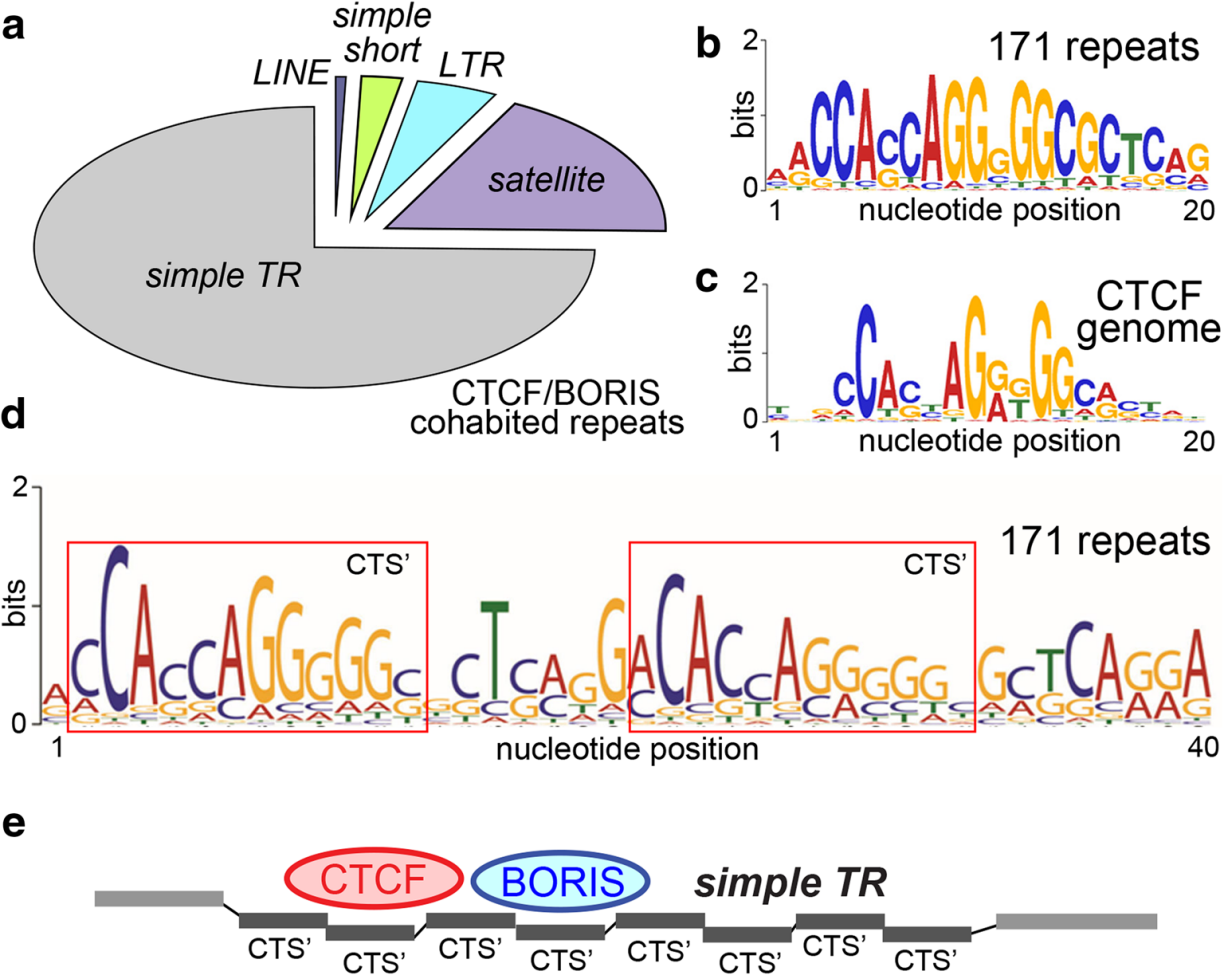
Distribution of repeat sequences and the co-binding consensus for BORIS and CTCFsites. **a** The chart showing the breakdown of repeat types among the features that are strongly bound (×4 enrichment or more) by both BORIS and CTCF, based on 171 ChIP-seq-validated repeats in Additional file 1: Table S2. **b** The co-binding DNA consensus derived from 171 co-bound repeats. **c** The whole-genome consensus for CTCF binding based on ChIP-seq data with the same parameters as in **b**. **d** The larger duplicate/staggered consensus for 171 repeats, when a 40-nucleotide window was interrogated. CTS’ denotes a short consensus for CTCF binding. **e** A model explaining the “staggered” emergence of cluster CTSs from tandem TRs containing CTS’

The simultaneous binding of BORIS and CTCF genome-wide in cancer cell lines was shown to reset, at least partially, the functions of CTSs in transcriptional regulation in accordance with germline-like program [55]. Thus, from the standpoint of cancer biology, it was important to characterize repeats bound by both CTCF and BORIS (CTCF and BORIS repeats, Additional file 1: Table S2), especially as they outnumbered other classes (Fig. 1e). The 171 distinct repeats in the CTCF and BORIS class were mostly represented by uncharacterized simple repeats, which can also be classified as VNTRs, and a small fraction of TEs, with the telomeric satellite *TAR1* notably dominating the rest of the group (Fig. 2a; Additional file 1: Table S2). It has to be appreciated that there is no certain way to determine whether both CTCF and BORIS co-bind the given individual repeat sequence, due to the multiple copies of repeats present and the propensity of CTCF and BORIS to induce interchromatin contacts [49, 55]. Nevertheless, the presence of cluster CTS is a strong indication of co-binding [55]. While this group included simple repeats long enough to harbor a single CTS, a more peculiar repeat type dominated this group. Namely, while a conventional 20-nucleotide GC-rich signature sequence was readily derived for the group as a whole, consistent with the CTCF-binding motif generated for the whole genome (Fig. 2b, c), a longer consensus, which is more in line with the span of the actual CTCF binding [67], showed that a duplication of a shorter binding signature (denoted CTS′) is present in these repeats (Fig. 2d). Thus, while an individual repeat unit does not enclose a bona fide cluster CTS, the tandem arrangement of this class sets a potentially multiple/staggered binding mode for CTCF and BORIS at these elements potentially generating a cluster site, if the tandem structure is long enough (Fig. 2e). Therefore, we can hypothesize that co-binding of CTCF and BORIS to the same site, as in this group of repeats, is facilitated when two binding regions are juxtaposed in cis, as happens in the rest of the genome [55]. The fact that multiple uncharacterized simple repeats were found in this class indicates that these elements should have a regulatory function in the epigenome mediated by dual binding by CTCF and BORIS.

Analysis of CTCF and BORIS co-binding at repeated DNA would have been incomplete without assessing the least characterized region of human epigenome—the chromatin of nucleolar organizer (NOR, or rDNA repeats). The bona fide human genomic rDNA has a very complex structure with multiple intervening sequences [68], and the NOR sequence from any human chromosome still remains to be determined. Therefore, human rDNA was not represented at TRF database and was not present on our microarrays. While we did not validate rDNA binding by CTCF and BORIS in ChIP-chip, it is known that the repeat unit contains a strong hotspot for CTCF binding facilitating CTCF’s interaction with PolI transcription machinery [69]. We used a “consensus” human rDNA repeat, as in [65], to align ChIP-seq reads and assess the potential differences between CTCF and BORIS binding (Additional file 2: Figure S2B). Comparing BORIS and CTCF binding showed that CTCF has a single binding site upstream of rDNA PolI promoter, consistent with published data in mice [70]. At the same time, BORIS appeared to have some enrichment at additional sites (Additional file 2: Figure S2A). These locations, however, corresponded to low-complexity regions (Additional file 3: Table S1), which were also present elsewhere in the genome. Unlike the established CTCF binding site, the two selected BORIS sequences that appeared to be enriched in ChIP-seq were not confirmed to bind BORIS by EMSA in vitro (Additional file 2: Figure S2C). Thus, one may assume that such sites likely represent an artifact of short reads’ alignment to tandemly repeated DNA, and the additional such sites were not tested. The presence of BORIS at the main Pol I regulatory site in rDNA, however, indicates that BORIS might be involved in ribosome biogenesis in cancer cells by virtue of co-regulating the rDNA transcription with CTCF.

### CTCF-only enrichment is found in older repeat classes

**Fig. 3:**
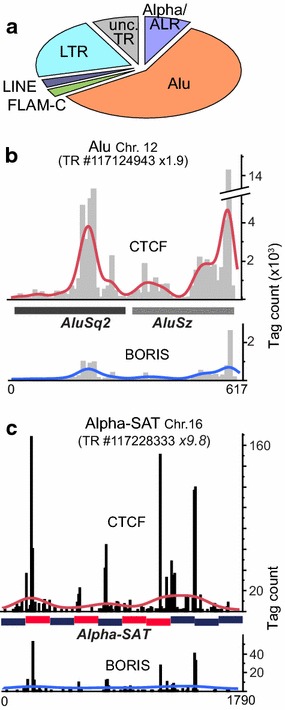
Repeats preferentially bound by CTCF are comprised mostly of evolutionary older high-copy repeats. **a** A chart of repeat types from Additional file 1: Table S2 that are strongly bound by CTCF (×4 enrichment or more by ChIP-chip) but are not enriched for BORIS (×1 enrichment or less). **b** An example of the distribution of ChIP-seq tag enrichment for CTCF and BORIS at a tandem repeat of two different Alu (shown in the schematic). The centers of normalized counts were binned along the DNA sequence (*histogram bars*) with the fit line applied accordingly. **c** An example of ChIP-seq tag enrichment distribution for BORIS and CTCF at an intrachromosomal run of ~10 alpha-satellites (CENP-B box containing repeats are colored *red*). The *histogram bars* and smooth line—as in **b**

The CTCF-only binding sites have a still unknown function in the genome, possibly unrelated to transcription [55]. PCA results in Fig. 1e enabled us to separate the CTCF-bound repeats that were refractory to BORIS intrusion (Fig. 3a). Thirty-eight individual CTCF-only repeats in this group were validated by ChIP-seq (Additional file 2: Table S2). This set includes major known types of repeats with long evolutionary history, while evolutionary young and simple TRs were largely absent. This agrees well with the studies, indicating that some CTCF-only binding sites in repeats are conserved in evolution [67]. Two examples of ChIP-seq analysis for repeats in this class, a TR of two Alu elements (Fig. 3b) and a run of divergent centromeric alpha-satellites (Fig. 3c), showed a robust enrichment by CTCF as compared to BORIS. As the enrichment of alpha-satellites by CTCF did not appear to be very strong, it is possible that a substantial fraction of alphoid elements in the K562 genome are not occupied by CTCF. Combined with the fact that CTCF binding does not appear to be correlating with CENP-B box presence (Fig. 3c), this may even indicate that only non-centromeric alpha-satellites are bound by CTCF. The absence of strong BORIS binding to this group of repeats agrees well with the underrepresentation of clustered CTS consensuses in this repeat group (not shown).

### A movable and evolutionary youngest class of TEs is specifically enriched in BORIS binding

**Fig. 4:**
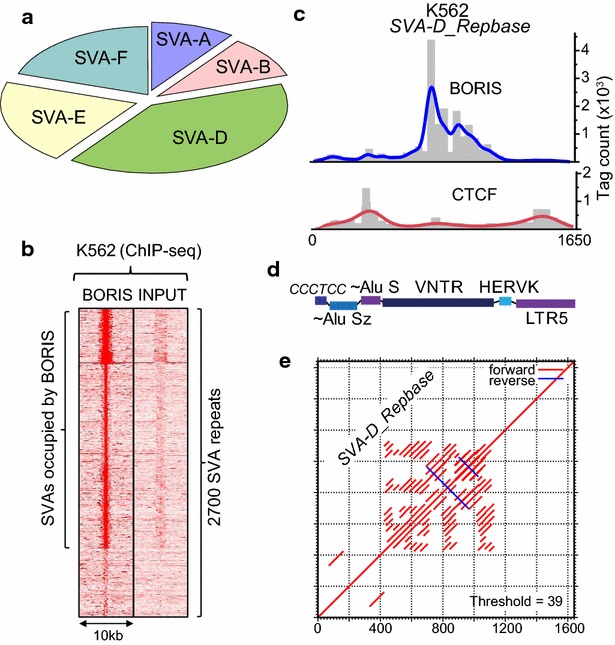
SVA repeats are preferentially bound by BORIS with a strong preference for VNTR region. **a** The chart showing the dominance of SVA elements among the repeats from Additional file 1: Table S2 that are strongly bound by BORIS (×4 enrichment or more by ChIP-chip) with no enrichment for CTCF (×1 or less). **b** BORIS occupancy is associated with SVAs repeats in K562 cells. The heatmap demonstrates the ChIP-seq enrichment of BORIS occupancy at SVAs element in K562 cells compared to input. The tag density was subjected to *k*-means ranked clustering with four clusters expected. **c** The ChIP-seq tag density distribution for a “canonical” full-length SVA-D element from Repbase shows that BORIS is clustered at the center of the element in a pattern complementary to CTCF. The normalized counts were binned along the DNA sequence (*histogram bars*) with the fit line applied accordingly. **d** The schematic structure of SVA-D element in **e**. **e** A diagonal alignment plot of DNA sequence for SVA-D in **c**, **d** indicating that BORIS enrichment corresponds to the VNTR region

The BORIS-only repeats, where BORIS binds without the equivalent presence of CTCF, are the most revealing with respect to BORIS biology in cancer cells, as they are directly involved in the transcriptional regulation of the non-repeated part of the genome [55]. Remarkably, in this group, the only 10 TRF classes that were validated fell within a single repeat type: the SVA family (Fig. 4a; Additional file 1: Table S2). The SVA repeats are a hominid-specific family, which is still currently mobile in the human genome owing to L1 activity [71, 72]. Overall, ChIP-seq analysis indicates that as much as 70 % of SVA elements could be occupied by BORIS in K562 (Fig. 4b). When this preference for SVA repeats was dissected for individual genomic repeat sequences, it became apparent that the enrichment by BORIS peaked in the central part of the element composed of the GC-rich VNTRs (Fig. 4c–e). VNTRs in SVA are GC-rich sequences with unknown molecular function. The patterns of CTCF and BORIS occupancy at SVA elements were distinct (Fig. 4c), unlike in other elements analyzed in Fig. 3. This might indicate the exceptional specialization of the VNTRs for BORIS binding in cancer cells. In order to exclude the possibility that SVA enrichment by BORIS is a specific property of K562, myeloid cells, or the female epigenome in general, we conducted ChIP-seq analysis of an unrelated cancer cell line with aberrantly activated BORIS, Delta-47 cells [55]. Although the difference between BORIS and CTCF enrichment was not as dramatic as in K562, the preference of BORIS was evident (Additional file 4: Figure S1A), notwithstanding the lower level of BORIS in Delta-47 [55]. Considering that the SVA’s VNTRs are dynamic in number and composition themselves [73], the finding of a global regulator BORIS bound to a mobile and extremely variable repeat class could be indicative of an additional germline-specific function of BORIS.

**Fig. 5:**
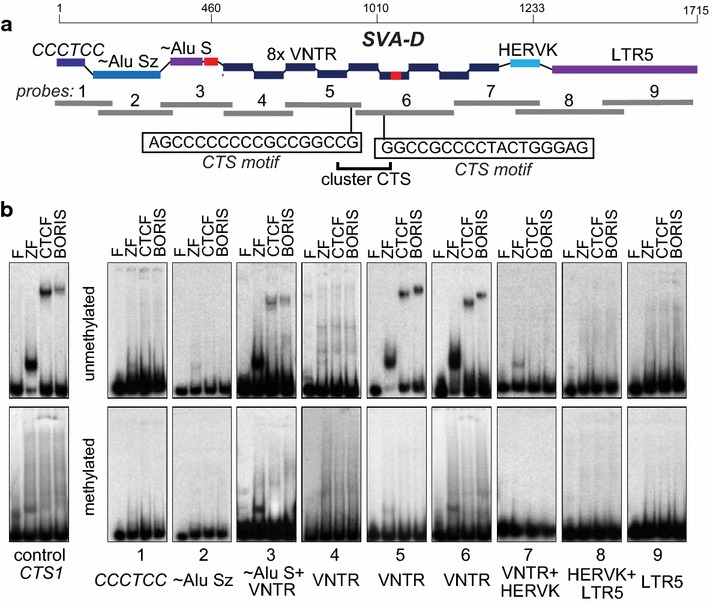
BORIS binding to SVA elements in vitro is based on binding to sequences in the VNTR. **a** The design and positions of EMSA probes based on a test-case SVA-D element (hg19, chr11:107782497–107784211). The two CTCF-binding motif-matching sequences are shown in probe 5 (MEME *p* value 7.95e−06) and probe 6 (*p* value 1.32e−05). *Red bars* denote two unique sequences acquired by this retrotransposon. **b** EMSA shows that the tested SVA-D element contains at least two distinct BORIS and CTCF binding sites in the VNTR region, with binding sensitive to 5metC methylation in vitro. The binding in probe 3 was shown to be due to the presence of a unique sequence

In order to map the locations of BORIS binding sites in SVA elements with higher precision, we designed nine probes corresponding together to a full-size SVA-D element (Fig. 5a) and analyzed them by EMSA with BORIS and CTCF proteins produced by in vitro translation. EMSA assay showed that the weak binding found in the AluS part can be attributed to a short unique sequence there (Fig. 5b). The central core of VNTR region, represented by two probes (5 and 6) in an EMSA, showed reproducible binding to both BORIS and CTCF proteins (Fig. 5b). Based on the EMSA data and CTCF motif analysis (Fig. 5b), these two VNTR sites juxtaposed to each other together form a cluster CTS, which is required for BORIS-only binding [55]. The 83-bp unique sequence embedded in the probe 6 in Fig. 5 was by itself unable to bind either protein (not shown). Not surprisingly, no discernible difference was detected between CTCF and BORIS in binding in vitro (Fig. 5b). This indicates that the BORIS' preference for SVA binding observed in chromatin (ChIP data) is likely determined by epigenetic factors. As CTCF is known to have both DNA methylation-sensitive and methylation-insensitive binding sites, we verified whether BORIS is able to bind VNTRs when CpGs are methylated. EMSA analysis with methylated probes (Additional file 4: Figure S1B) showed that both CTCF and BORIS binding were abolished by full CpG methylation (Fig. 5b). This likely indicates that the preference of these sites for BORIS binding in chromatin, even if partially controlled by DNA methylation, must be fine-tuned with respect to specific CpGs methylation.

What could be BORIS activity at SVA elements? Our previous results on the genome-wide consequences of modulation of BORIS expression indicated that BORIS could serve as an activator as well as repressor [55]. The distinct preference of the aberrantly expressed BORIS for SVA elements may potentially indicate that BORIS has some regulatory activity at these elements in germline and/or in cancer cells. As there is little doubt that SVAs mobilization is detrimental to genome stability, because they are under a strong repression in primates [73–76], a possible BORIS involvement in the regulation of SVA transcription must be biologically important. Indeed, the transcription is required for SVA transposition, and it could also have a regulatory role in the expression of neighboring genes.

### BORIS acts as a transcriptional co-repressor of a significant proportion of SVAs in K562 cells

While the transcription unit of SVAs is not well characterized [76, 77], the Alu-derived sequences are the chief drivers of transposition in SVA [78]. Thus, SVAs contain sequences potentially transcribed by both RNA Pol III and Pol II, either of which can drive retrotransposition [79]. At the same time, based on structural considerations, it is unlikely that SVA elements are actually transcribed by Pol III [77]. We tested whether there was a difference in the occupancy of RNA Pol III factors at SVA elements between the publicly available ChIP-seq datasets for BORIS-positive K562 and BORIS-negative NHEK. Incidentally, we found no notable enrichment at any SVA elements for POLR3G, BDP1, BRF1, BRF2, or RPC155 (data not shown).

**Fig. 6:**
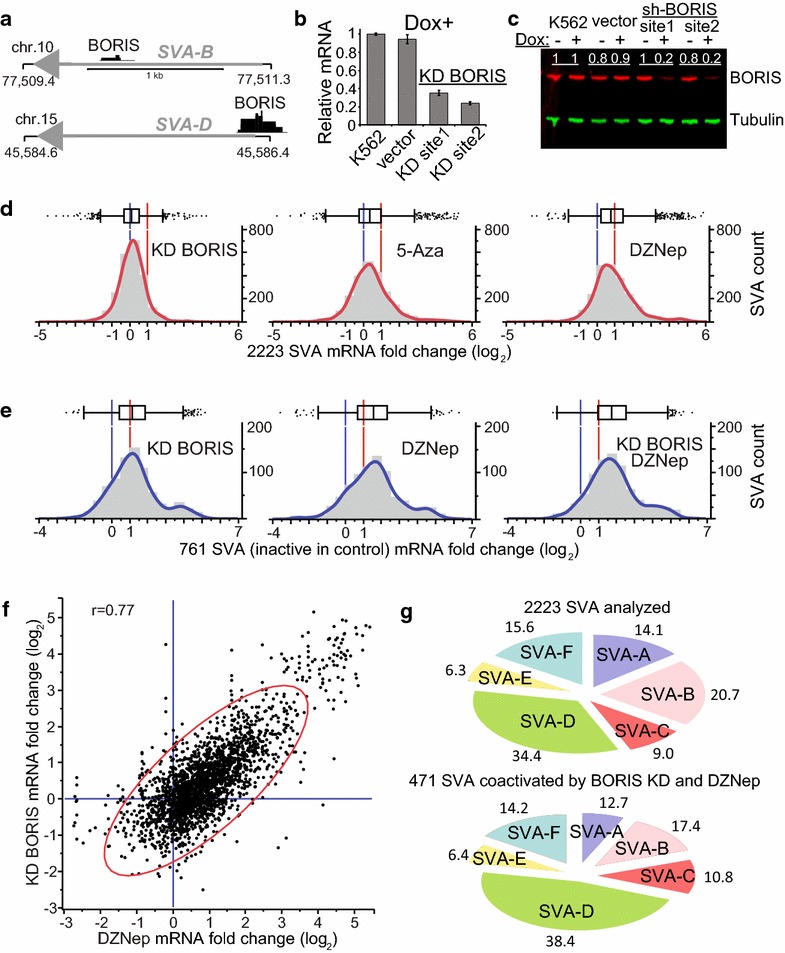
Downregulation of BORIS and epigenetic remodeling show concordant activation of SVA transcription in K562 cells. **a** The two SVA elements with highest expression in testis with the position of BORIS ChIP-seq peaks in K562. The absence of strong BORIS binding indicates that BORIS is unlikely to act as SVA activator in testis. Genomic coordinates are in kb. The SVA-D shown is intergenic, while the SVA-B is antisense intronic. **b** RT-qPCR shows the downregulation of BORIS in K562 clones with stable integration of Tet-On inducible anti-BORIS shRNA constructs (site 1 and site 2), 48 h after shRNA induction. Un-infected K562 cells and a clone with the integrated empty vector were controls. Only the experiments in the presence of doxycycline (Dox+) are shown. **c** Immunoblotting with anti-BORIS mAbs demonstrates fourfold–fivefold depletion of BORIS protein in shRNA clones. The quantification of relative BORIS amount (*white numbers*) was performed using LiCor software and alpha-tubulin as a reference. **d** RNA-seq analysis of differential expression of 2223 SVA elements longer than 1 Kb mapped in the human genome versus K562 infected with the empty vector (SVAs that were constitutively silent were not included). Shown are the distributions of ratios of RNA-seq difference in: BORIS KD K562 cells (paired two-tailed *t* test *p* value <0.001), K562 treated with 5-AzadCyD (5Aza) (paired two-tailed *t* test *p* value <0.001), and K562 treated with DZNep (paired two-tailed *t* test *p* value = 0.001). The RNA-seq reads enrichments for SVA elements were normalized to the total number of reads in each individual experiment. The mean and standard deviation diagrams are shown on *top* of each graph. The graphs demonstrate the overall increase in the shift toward higher SVA expression from BORIS KD to 5Aza and especially DZNep treatments. *Vertical blue lines* correspond to the unchanged expression over control, *red*—to twofold increase. **e** The RNA-seq analysis of BORIS KD, DZNep treatment, and the combination of both on the transcription of SVA elements that were apparently silent in control experiments (i.e., <10 normalized counts with over twofold increase by any treatment) shows a reproducible compound effect of BORIS KD and DZNep treatment on SVA activation (for the latter, the whole 2223 SVA sample’s paired two-tailed *t* test *p* value <0.001). The corresponding means of the distributions with standard errors of the mean: KD 1.03 ± 0.03, DZNep 1.37 ± 0.03, KD and DZNep 1.55 ± 0.02. **f** *Dot*-*plot* of RNA-seq normalized counts of BORIS KD versus DZNep treatment of K562, expressed as fold enrichment over the empty vector control. Only the SVA elements that were silent in the control are included. The *blue lines* correspond to cutoffs with no change in expression. **g** SVA elements that show concordant activation by BORIS KD and DZNep treatment do not belong to a preferential SVA class. The *pie diagrams* show the breakdown of SVA classes among all 2223 elements included in the analysis and among 471 elements co-activated by both treatments

Next, we focused on the RNA Pol II transcription of SVAs and first took advantage of CAGE datasets available for K562 (BORIS positive) and NHEK (BORIS negative). The CAGE reads were aligned to the genome, and the extended areas corresponding to SVA elements were analyzed separately. However, the levels of SVA transcription were low, and SVA transcription in BORIS-positive K562 cells was mostly well correlated with the BORIS-negative NHEK cells (Pearson correlation 0.98). At the same time, RNA-seq data available for human testis suggest that some SVA elements could be highly expressed; however, the two full-length (FL) SVA elements with highest expression in human testis showed no ChIP-seq enrichment for BORIS at the VNTRs (Fig. 6a). The extension of analysis in Fig. 6a to 59 additional SVA elements with various degrees of BORIS occupancy showed only marginal levels of expression without any correlation with BORIS presence at the VNTRs (not shown). Thus, it is highly unlikely that BORIS bound to VNTRs serves as a transcription activator of SVA transcription in K562 cells.

At this point, one may hypothesize that the affinity of BORIS to VNTRs of SVA elements demonstrated in K562 is a reflection of its role in germline pertaining to these elements and that this role is likely a repressive one. Indeed, we recently showed that despite BORIS previously perceived as an activator, BORIS upregulation was linked to the repression of some genes and, vice versa, BORIS downregulation has resulted in some gene being activated [55]. Therefore, we investigated the K562 cells with downregulated BORIS. As SVA elements might be rapidly repressed by some other mechanism in the absence of BORIS, we could not rely on BORIS KO data [55], as the points of comparison there were separated by a long period of time. Instead, we experimented with the downregulation of BORIS expression in K562 cells for a short period of time using inducible shRNA. This approach enabled us to assess immediate downstream effects of BORIS downregulation. We constructed K562 cell lines with two alternative inducible anti-BORIS shRNA constructs stably integrated into the genome and conducted RNA-seq experiments after BORIS KD for 48 h. Neither the degree of BORIS depletion nor the time span of the experiment was sufficient to induce the differentiation, as was described for BORIS KO [55]. While genome-wide expression of genes responding to BORIS KD was almost evenly divided between up- and downregulation of transcription (data not shown), SVA elements longer than 1 kb were notably activated (Fig. 6d). In order to address whether any SVA were actually downregulated upon BORIS KD, we isolated the subclass of SVA elements that were already expressed in K562 and compared their expression to BORIS KD cells. As shown in Additional file 5: Figure S3A, the 70 SVA elements that were expressed did not significantly change their expression upon the downregulation of BORIS.

In order to understand better the nature of SVA activation, we treated control K562 cells with 5-AzadCyD (5-Aza-2′-deoxycytidine), an inhibitor of DNA methylation [27, 80–82], and DZNep (3-deazaneplanocin A), which indirectly suppresses EZH2 that catalyzes histone H3 lysine 27 methylation [83, 84]. Both drugs result in the removal of inhibitory epigenetic marks from DNA and chromatin, respectively. RNA-seq analysis of K562 cells treated with these DNA methylation or H3K27me3 inhibitors indicated that SVA elements that were already active were upregulated slightly (Additional file 5: Figure S3B, S3C), while the group as a whole was preferentially activated. The 5-AzadCyD effect was similar to BORIS KD, and the DZNep effect was more pronounced (Fig. 6d). Thus, we next asked whether these treatments could be preferentially affecting the same subset of SVA elements as BORIS KD or a distinct one. Using the DZNep treatment as an example, Fig. 6e, f, we showed that BORIS KD largely acted concordantly with DZNep (correlation 0.77) to activate SVA transcription of the elements that were silent in the control. It was also evident that the BORIS KD-dependent activation was not specific to any particular subclass of SVA repeats (Fig. 6g), indicative of a common pathway.

### A distinct type of BORIS function at the SVA-F1 TEs

**Fig. 7:**
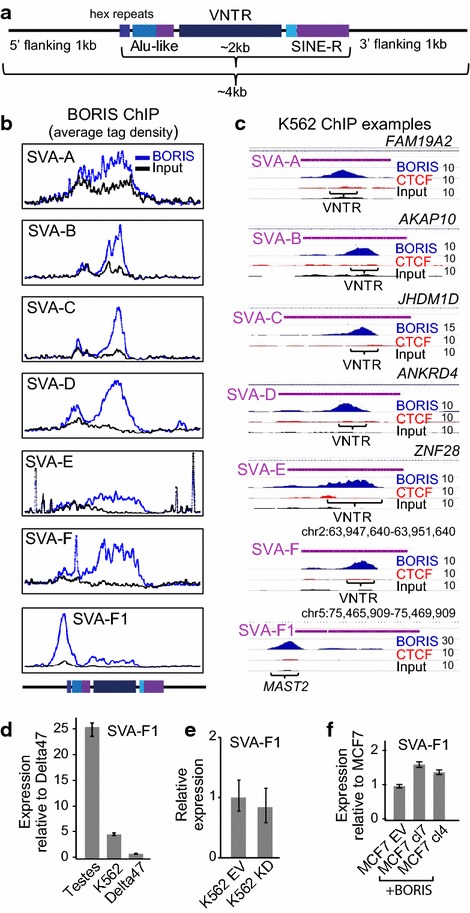
BORIS is enriched at the 5′-transduced sequence at the SVA-F1 family repeats. **a** A schematic of tested ~4 kb sequence encompassing SVAs elements. The sequences were used to plot the average tag density of BORIS ChIP-seq in K562 cells. **b** BORIS is predominantly associated with VNTR repeats of SVAs, with the exception of SVA-F1 where BORIS is bound at the 5′-transduced sequence. The average tag density (tags/ten million) is shown for BORIS versus the input of ChIP-Seq data in K562 cells. **c** The individual genomic examples of BORIS and CTCF occupancy corresponding to **b**. Data were normalized to the number of mapped reads and the number of SVA elements. **d** SVA-F1 expression is upregulated in testis. qRT-PCR data on total testis mRNA and two BORIS-positive cancer cell lines. **e** KD of BORIS in K562 does not change the SVA-F1 expression. qRT-PCR data of BORIS KD by shRNA relatively to the control empty vector (EV); SVA-F1 primers were from [88]. **f** The induced BORIS expression in MCF7 cells slightly upregulates SVA-F1 expression. qRT-PCR for two clones expressing ectopic BORIS [55] is compared to MCF7 transfected with the vector only (EV)

The prevalent repressive role of BORIS on SVAs does not exclude the possibility that under certain conditions it could actually serve as an SVA activator. One such case could be the *MAST2*/SVA-F exon trap [85–87]. The capturing of *MAST2* sequence by SVA-F resulted in the formation of a novel family (SVA-F1), represented by 81 members in the hg19 human genome assembly [85, 88] The 5′ flanking region of SVA-F1 family is the result of a fusion between the first exon of *MAST2*, a gene expressed in testes, with the SVA-F repeat. Thus, it is conceivable that in testis BORIS acts as an activator of SVA-F1. This is possible as the binding of BORIS to SVA-A through SVA-F is within the VNTR region, but for SVA-F1 BORIS preferentially binds within the 5′ flanking region of the SVAs, upstream of the hexamer repeat region (Fig. 7a–c). It is worth noting that the first exon of *MAST2* is not just occupied by BORIS in K562 cells but is also aberrantly expressed in cancer cells together with BORIS expression (Additional file 6: Figure S4A). Thus, BORIS binding outside of SVA elements may serve as an external promoter for SVA-F1 expression. The numbers of nucleotides captured from the *MAST2* exon by SVA-F1 vary from 36 to 382, with potentially four BORIS binding sites incorporated into 382 bp-promoter sequence (Additional file 6: Figure S4B). That may create a possibility for multiple TSSs starting from any of four BORIS binding sites. It may also explain the presence of *MAST2* SVA-F1 sequences of varying length. Indeed, the common feature of nearly all SVA-F1 transduced sequences is the presence of at least one BORIS binding site. In agreement with multiple BORIS binding sites in the transduced sequence the BORIS occupancy significantly correlates with the length of transduced sequence (Additional file 6: Figure S4B). While SVA-F1 sequences are strongly expressed in testis, they remain methylated in other instances of substantial hypomethylation of the genome [89]. Their expression is also quite low in BORIS-positive cell lines (Fig. 7d). Neither did the KO of BORIS in K562 cells change the overall expression of SVA-F1 (Fig. 7e). Nevertheless, the ectopic BORIS expression in BORIS-negative cells appears to have a slight activating effect on SVA-F1 (Fig. 7f). We also analyzed the putative promoter-trapping events similar to the *MAST2* case throughout human genome and identified several putative locations of such occurrences. For example, we found that *NDUFV2*, *FDX1*, *PHKA1*, *WDR33*, *RHOT1*, *ZNF488*, *ZNF487*, *PHLPP2*, *TOM1L2*, *ARL4A*, and *MPPE1* promoters were trapped by SVA repeats and used for SVA expression in K562 cells (Additional file 7: Figure S5; Additional file 8: Table S3). One of the common features of all these promoters is the presence of BORIS binding sites inside the trapped sequences, occupied by BORIS in K562 cells and transcribed in BORIS-positive cells (Additional file 6: Figure S4; Additional file 7: Figure S5). Based on such data, one would be compelled to conclude that the capture of BORIS binding sites by SVAs is beneficial for their transcription. The trapping of BORIS binding sites within the promoter region of SVA repeats may also be indicative of an existing pathway for non-random SVA integration.

In conclusion, it appears that BORIS acts as a co-repressor of SVA transcription in K562 cells, alongside DNA methylation and heterochromatinization. It is therefore likely that BORIS plays a similar role in the germline, with the exception of promoter-trapping events. These findings indicate a potential biological role of BORIS as a regulator of active TEs in human genome.

## Discussion

The “explosive” chromosome instability is confirmed to be one of the defining features of cancer genome [90, 91]. This notion has sparked multiple attempts to find either a unifying mechanism or a set of concurrent mechanisms for this process [92, 93]. The early onset of chromosome instability in cancer and pre-cancer cells strongly indicates the epigenetic roots of the destabilization. In this context, the roles of chromatin states of genomic repeats in cancer are of significant interest because they directly bridge the epigenetic landscape with a potential to destabilize genome via transposition and/or recombination. TEs that can pose a danger to genome integrity tend to be silenced for recombination and retrotransposition by epigenetic mechanisms [17, 73, 94]. Here, we found evidence of BORIS involvement in the co-regulation of TEs. The established role of BORIS as a transcriptional regulator in cancer [55, 95] and as activator of testis-specific genes [70, 96, 97] might also be applicable to the states of genomic repeats in cancer cells. Nevertheless, the role of BORIS with respect to genomic repeats was previously totally unknown, despite the significant recent progress in understanding the transposition as the primary venue of genome evolution pertaining to the distribution of CTCF binding sites [67].

In this study, we established that BORIS, upon its activation at a relatively high level in cancer cells, has a substantial capacity to occupy the same sites in the repeated elements as CTCF (Fig. 1e). We can presume, with a high level of certainty, that it is a manifestation of the BORIS’ co-functions with CTCF in the normal germline [55, 70]. While co-binding is generally expected due to the DNA-binding properties of the two proteins in vitro, the recent discovery of cluster sites being a prerequisite for CTCF and BORIS co-binding or binding of BORIS alone [55] suggests that a significant fraction of such repeats have cluster site configuration. Indeed, the assessment of DNA consensus characteristic for BORIS and CTCF co-bound repeat sites (Fig. 2c) showed no significant deviation from the basic unit of CTCF consensus derived from the genome-wide binding studies (Fig. 2b), but revealed the presence of a staggered arrangement (Fig. 2d), which potentially enables such TR locations to become super-cluster sites with ample co-binding capacity. The characterization of repeats that are co-occupied by CTCF and BORIS showed that the bulk of co-binding seems to be associated with the low-copy simple TRs (Fig. 2a). These elements have a relatively narrow length distribution, most are longer that 50 nt, indicating that they are under selection, possibly by the requirement to bind CTCF or BORIS. While expansion of short TRs is known to cause disease in a number of studied cases [98, 99], their genome-wide biological role is obscure. Thus, it is likely that BORIS and CTCF co-binding there uncovered a putative regulatory role for these elements in germline and/or cancer transcription.

The few repeat types that show a significant bias toward CTCF-only binding are rather enigmatic, as the function of CTCF-only sites genome wide is not well characterized [55]. The most notable case here is the centromeric repeats, where recombination is highly undesirable [100], but the transcription was nevertheless found to be of paramount importance for normal kinetochore formation [101]. While CTCF’s binding at alpha-satellites and its involvement in centromeric transcription were not studied, the interaction between CTCF and some centromeric proteins has been invoked at ectopic sites [102].

The most distinctive result generated by this study is the high preference of SVA repeats for BORIS binding, as compared to binding by CTCF in K562 (Fig. 4). Unfortunately, in the absence of ChIP data for BORIS from human testis one cannot be absolutely sure that it is also the situation in normal testis. The functions for SVA that are described so far are attributed to the disruption/features of insertion sites rather than to the transcription originating within the insertion [103, 104]; yet the finding of BORIS binding hints at the regulatory role of SVA VNTRs themselves. The presence of several BORIS binding sites within the VNTR repeats (Figs. 4c, f, 5), which are actually required for SVA transposition [78], indicates that the BORIS protein and SVA elements may have even undergone co-evolution, as has been recently suggested for other ZF proteins [73]. Thus, one may expect the SVA elements to play a notable regulatory role in germline development and genome evolution in primates. In that regard, the recent studies on gibbon genome [2, 105] provided some invaluable insight into the new level of plasticity that SVA-like elements LAVA infused into primate genomes. At present, one cannot conclude whether SVA TEs merely represent a genetic load or actually have a physiological role in germline. Despite human SVAs being associated with at least some chromosomal breaks [106], we could probably exclude the direct contribution of SVA elements into the meiotic recombination, as DSB maps of human meiosis [107] did not correspond to SVA locations (not shown).

By applying RNA-seq analyses to the K562 cells, we found a strong evidence of a substantial fraction of SVA elements being transcriptionally activated upon BORIS KD (Fig. 6d–f). This was a strong indication that BORIS acted as a repressor of SVA transcription for that repeat group. This conclusion is further reinforced by the finding that this repressive activity is additive with DNA methylation and with the formation of repressive chromatin structure (Fig. 6e, f). Therefore, we could conclude that BORIS participates in the repression of SVA elements that are located in the heterochromatin-like regions of epigenome. This BORIS-mediated tier of SVA repression could have an exceptional significance in male germline, where the rounds of DNA demethylation [108] could potentially open SVA retrotransposons for a transient activation leading to germline mutations, as it has been found in pluripotent cells [109].

The addition of BORIS to cancer cells’ chromatin constitutes a potent epimutation, as it could introduce a substantial change into CTCF’s functions [36]. Some of these changes were recently documented, particularly with respect of recapitulating the germline pattern of gene regulation [55]. With respect to the genomic repeats, the associated rewiring of epigenetic regulatory network, which is normally embodied by CTCF alone in somatic cells, may greatly alter the functional role of inserted repeats themselves, e.g., their expression and transposition, as well as their propensity to regulate neighboring genes and chromatin domains.

## Conclusions

As a result of this study, by employing ChIP-chip and ChIP-seq approaches, we characterized CTCF and BORIS binding patterns of genomic repeat binding upon aberrant BORIS expression in the K562 cancer cell line, which is dependent on BORIS for proliferation. This study showed that, while CTCF-only enrichment is found in most known repeat classes, BORIS and CTCF bind together predominately to the uncharacterized simple TRs, which likely form compound cluster binding sites. We discovered that the SVA elements, a presently active family of TEs in human genome with a strong mutagenic potential and a role in transcription regulation, are specifically enriched in BORIS, with binding concentrated at the VNTR region. Furthermore, RNA-seq analysis of BORIS KD in K562 showed that BORIS acts to repress multiple SVA, alongside the transcriptionally repressive histone modification and DNA methylation. These finding uncovered a novel function of BORIS in controlling the levels of TE transcription in cancer cells and likely in the germline.

## Methods

### Cell culture, transfection, and lentiviral infection

K562, Delta-47, and HL60 cell lines were grown in IMDM (Hyclone) supplemented with 10 or 20 % Tet-approved-FBS. HEK293T/17 cell line was grown in DMEM (Hyclone) supplemented with 10 % FBS. Transfection was done according to manufacturer’s instructions using X-tremeGENE 9 DNA Transfection Reagent (Roche). To package lentivirus, HEK293T/17 cells were cotransfected with the vector Tet-pLKO-Neo (Addgene) or anti-BORIS shRNA derivatives and two packaged plasmids psPAX2 and Pmd2.G. Lentivirus stocks were collected 72 h post-transfection and used to infect K562 at 40–50 % confluence using 500 µl lentivirus stock and 8 µg/ml polybrene (Sigma). The media were then changed 12 h after infection to include 600 µg/ml G418, and the cells were selected for G418 resistance for at least 4 weeks. The resistant clones were selected in 96-well plates and analyzed by RT-qPCR and immunoblotting. The stable clones were induced by 200 ng/ml doxycycline to activate the Tet-On promoter.

### The tiling repeat microarray

The design for this custom array [65] was conducted at Roche/Nimblegen using tiling approach. As a source for the design, we used a catalogue of human TRs generated by TR finder [110, 111]. The version of TRF algorithm used for the design of the array generated 947,696 distinct repeat instances based on the human genome. The tentative estimate of redundancy conducted by applying the most stringent versions of TRF suggests that the repeat dataset had about 40 % sequence redundancy. The repeats were broken into 50-base tiles using the following rules: Tiles were picked based on the predicted hybridization normalization; when the repeat was shorter than 50 nucleotides, it was extended in tandem fashion. Our tiling approach has generated some additional redundancy within tiles themselves because long homogeneous repeats produced a number of identical tiles. The redundancies within the array did not interfere with microarray data analysis, as the primary hybridization signal was recorded for each tile independently of any other. The final array design contained 2,166,672 features, including two control sets: 29,161 random sequence tiles and 181 tiles from the rDNA locus of *Saccharomyces cerevisiae*.

### ChIP-chip and ChIP-seq

For the ChIP-chip and ChIP-seq, anti-CTCF and anti-BORIS ChIP were conducted from at least 50 million cells growing asynchronously. ChIP-seq preparation and analysis were done essentially as described in [55]. The specificity of ChIP reactions was validated by qPCR for known targets: the *TSP50* and *CST* promoters for BORIS, and the MYC promoter sites for CTCF as in [96, 97].

For ChIP, cells growing asynchronously were cross-linked (10 min, 1 % formaldehyde, 23 °C) quenched for 10 min by 200 mM glycine, washed three times with PBS, and then resuspended in chromatin buffer (150 mM NaCl, 1 % Triton X100, 0.1 % SDS, 20 mM Tris–HCl pH8.0, and 2 mM EDTA). DNA was sheared using Covaris S220, so that most fragments were in the 300- to 500-bp range. Chromatin was immunoprecipitated overnight with magnetic beads (DiaMag, Diagenode, Inc.) loaded with anti-CTCF or anti-BORIS antibodies as described in [55]. The immunoprecipitate was washed, cross-links reversed, protein component was digested with proteinase K, and DNA was extracted using phenol/chloroform/isoamyl alcohol. DNA concentration was measured by Qubit (Life Technologies) and/or Nanodrop (Thermo Scientific) fluorimeters. For ChIP-chip, the immunoprecipitated DNA was amplified using the Phi29 strand-displacement procedure (GE Bioscience) following the concatemerization of precipitated DNA fragments via ligation to double-strand adaptors containing BamHI overhangs and internal SapI sites. Both amplified and non-amplified samples showed essentially the same relative enrichment for known sites of CTCF and BORIS binding. Following the amplification, adapters were removed by SapI digestion and agarose gel purification. Input DNA was used as a hybridization reference for the hybridization of amplified ChIP DNA to a set of custom TR arrays (Roche-Nimblegen). Raw intensities for each channel were centered against the mean of control features set, including random oligonucleotides and yeast rDNA. Then, Lowess smoothing was applied to two-channel data to generate corrected M values that were used in subsequent analyses. The Lowess normalization, SAM, and PCA calculations were done using publicly available R scripts. For downstream analysis of ChIP-seq data, the Illumina reads (50 bp) were aligned to human repeat subgenome generated by TRF [111] using BLAT [112] (allowing 95 % identity) and/or Bowtie [113] (with parameters -v 2 --best --strata --tryhard). seqMINER [114] was used to analyze and plot CAGE expression data from published datasets. Motif Elicitation (MEME) software [115] was used to derive consensus sequences from genomic repeats with parameters (-mod oops -revcomp -w 20) to identify motifs on both DNA strands.

### Analysis of public high-throughput genomic data

ENCODE/RIKEN data (GSE34448) for K562 and NHEK cell lines were used in this study. The DSB maps of human meiosis were derived from [107].

### Immunoblotting

Protein extracts were prepared by lysing cells SDS-PAGE sample buffer after washing with PBS supplemented with 1× protease inhibitor cocktail (Roche Applied Science). Protein samples were separated by SDS-PAGE, transferred to a PVDF membrane, and incubated with the appropriate primary antibodies, followed by detection using LiCor secondary antibodies fused to fluorochromes. Photoluminescent images were captured by scanning and processed for quantification using LiCor workstation.

### Immunofluorescent cell staining

K562 and HL60 cells were spun down in Cytospin centrifuge (Thermo Scientific) onto poly-Lysine-coated coverslips and fixed with 4 % paraformaldehyde for 10 min, followed by cold methanol for 10 min. Cells were permeabilized with 0.1 % Triton X-100/PBS for 10 min and then blocked with BSA for 30 min, after which they were incubated with primary antibodies. After washes, the anti-rabbit or anti-mouse secondary antibodies conjugated to either Alexa Fluor 647 or Alexa Fluor 488 were applied. Cells were mounted for microscopy in mounting media containing DAPI and images captured using either confocal (Zeiss) or wide-field (Olympus) inverted microscopes.

### Electrophoretic mobility shift assay (EMSA)

To map CTCF and BORIS binding sites in SVA repeats, the SVA subfamily D repeat (chr11: 107,782,495–107,784,189, GRCh37/hg19) was covered with nine overlapping DNA probes either amplified by PCR or synthesized as oligonucleotides (Additional file 3: Table S1). PCR amplified products were cloned into the pCR2.1 TOPO vector (Invitrogen), and the sequence was confirmed by DNA sequencing. DNA fragments were labeled with [γ-32P] ATP at the 5′ ends by T4 polynucleotide kinase per Invitrogen protocol. Labeled DNA fragments were gel purified, and equal amount of each fragment was used for EMSAs. FL human CTCF, 11ZF domain of CTCF, and FL human BORIS were synthesized from pCITE expression vectors (EMD Millipore), using the reticulocyte lysate-coupled in vitro transcription-translation system (TNT, Promega). Binding reactions for EMSA were for 1 h at 23 °C with 4 µl of in vitro synthesized DNA-binding proteins in binding buffer [25 mM HEPES pH7.6, 100 mM KCl, 2 mM MgCl2, 10 % glycerol, 0.5 µg poly(dIdC) × poly(dIdC)]. DNA–protein complexes were resolved on 5 % non-denaturing polyacrylamide gels in 0.5× Tris-borate-EDTA buffer. *Gal3ST1* promoter fragment was used in EMSA as a positive control for both CTCF and BORIS binding [97]. To test methylation sensitivity of protein binding, all labeled probes used in EMSA were methylated using SssI methyltransferase (New England BioLabs) by the following protocol: 200 ng of each oligonucleotide was combined with 2.7 μl of NEBuffer 2, 3 μl (12 U) of SssI methylase and 1 μl of *S*-adenosylmethionine (32 mM). After 3 h of incubation at 37 °C, 0.5 μl of NEBuffer 2, 3 µl (12 U) of SssI methylase, and 1 μl of *S*-adenosylmethionine (32 mM) were added, and the reaction incubated for an additional 3 h at 37 °C. The completion of methylation was assessed by digesting them with the methylation-sensitive enzyme AciI (Additional file 2: Figure S2B).

### RT-PCR and quantitative PCR

Total RNA was prepared using Trizol (Invitrogen). cDNA was prepared using the Primescript™ RT Reagent Kit with genomic DNA Eraser (perfect real time) (TaKaRa) according to the manufacturer’s protocol. Quantitative PCR (qPCR) was performed using SYBR Premix Ex Taq™ (TaKaRa) and the Mx30005P QPCR System (Agilent).

### RNA-seq analysis

For the RNA-seq experiments, inducible BORIS knock down (KD) and control cell lines were created by infecting K562 cells with 3 different Tet-on lentivirus constructs: empty vector pLKO-Tet-ON-neo [116], and two alternative anti-BORIS shRNA constructs. Several stable clones of each infected cell line were selected using 600 µg/ml G418. BORIS KD vectors were constructed to express the following shRNA templates: GGAAATACCACGATGCAAATT (Site 1) and GGTGTGAAATGCTCCTCAACA (Site 2). For lentivirus vectors construction, the annealed oligonucleotides were inserted into the pLKO-Tet-On-neo vector between AgeI and EcoRI restriction sites. After 72-h induction by doxycycline, BORIS mRNA was reproducibly showing 2.5-fold to threefold reduction, while BORIS protein levels were robustly decreased over fivefold (Fig. 6c, d). For RNA analysis, these K562-inducible stable shRNA cells were plated in 10-cm plates at 40–50 % confluence in DMEM media and left to grow in the presence of doxycycline (200 ng/ml) for 96 h. For the 5-aza-deoxycytidine and DZNep experiments, cells were identically pretreated with doxycycline, harvested, and re-plated at 50–60 % confluence to grow 48 h in the presence of either 500 nM 5-aza-2′-deoxycytidine, 1 µM DZNep or DMSO. The degree of genomic DNA demethylation was assessed using DNA IP with anti-5-methylcytosine mAb MABE146, clone 33D3 (EMD Millipore), and qPCR against known targets. The effectiveness of DZNep treatment was assessed by immunoblotting against the EZH2 protein with D2C9 rabbit mAb (Cell Signaling Technology). The cells were then collected, frozen, and outsourced for Illumina sequencing to RiboBio (Guangzhou). The amount of RNA submitted for each individual run was on average 85 µg (Nanodrop). The quality of RNA was assessed by the Agilent 2200 TapeStation. About 20 million reads were obtained for each individual experiment. Four biological replicates were produced and analyzed for each set of experimental conditions. The results of all RNA-seq experiments were analyzed for consistency and reproducibility using Cufflinks 2.0.0 [117] following reads alignment to the human reference genome (hg38) using TopHat2, with the default parameter setting. Upon that validation, for SVA alignment to RNA-seq data, a sub-genome file of 2223 SVA elements was assembled from elements mapped in hg38 that were longer than 1 kb, i.e., to ensure that VNTRs were included. The SVA elements were aligned to RNA-seq reads with Bowtie (-v0), and read counts per each element were normalized according to total read numbers in each experiment. Then, fold-enrichment ratios relative to the averaged normalized reads in the empty vector experiments were calculated.

## Additional files

10.1186/s13072-016-0084-2 Classes of repeats co-occupied or differentially occupied by CTCF and BORIS.
10.1186/s13072-016-0084-2 BORIS binds at the same regulatory site as CTCF in rDNA. (A) The distribution of Chip-seq tags across the consensus rDNA repeat is shown for the input, CTCF ChIP-seq and BORIS ChIP-seq. The sites chosen for EMSA are indicated with brackets. (B) The corresponding structure of “canonical” rDNA repeat. The long arrow corresponds to the Pol I transcript; the short arrow—noncoding RNA; NTS—non-transcribed spacers. (C) EMSA of the chosen rDNA sites confirming that the known CTCF site in PolI promoter is co-occupied by CTCF in BORIS, while sampling of BORIS-only putative sites shows that there is no BORIS binding to these sites in vitro. (D) The assessment of CpG methylation for probe #3 used in (C) using the diagnostic digestion with Aci I endonuclease, before and after methylation.
10.1186/s13072-016-0084-2 EMSA oligonucleotides.
10.1186/s13072-016-0084-2 Extended analyses of SVA-D. (A) BORIS binding peaks at SVA VNTRs in Delta-47 cells. The ChIP-seq tag density distribution for the full-length SVA-D element from Repbase indicates that BORIS retains preference for VNTR region event in this cell line completely unrelated to K562 and with a substantially lower BORIS expression level. The normalized counts were binned along the DNA sequence (histogram bars) with the smoothing line added. (B) The assessment if CpG methylation of oligonucleotides used in EMSA. DNA fragments were digested by the methylation-sensitive endonuclease Aci I before and after methylation.
10.1186/s13072-016-0084-2 The expression of SVA elements that are transcribed in K562 cells is not affected by BORIS dosage. RNA-seq differential ratio distribution for 75 SVA elements, which were apparently transcriptionally active in the untreated K562 cells (i.e., over 10 normalized counts in empty vector control). Only elements longer than 1 Kb were included in analysis. Shown are the graphs for BORIS KD K562 cells, K562 treated with DZNep and the combination of both treatments.
10.1186/s13072-016-0084-2 SVA elements capture BORIS binding sites from unique gene promoters. (A) BORIS ChIP-seq and deep CAGE (Cap Analysis of Gene Expression)-seq (ENCODE data) coverage tracks for the *MAST2* gene (upper track) and for the SVA-F1 element (lower track) in K562 cells. BORIS occupancy at the MAST2 first exon sequence coincided with the multiple transcription start sites (TSS) for *MAST2* and SVA-F1 family expression in K562 cells. The black arrows show the direction of transcription based on CAGEs enrichment on plus strand. The red double-headed arrows show the *MAST2* sequence captured by SVA-F1 family from the *MAST2* gene. (B) ChIP-seq enrichment of BORIS occupancy depends on the number of BORIS binding sites in the transduced sequences. The top panel is the schematic representation of SVA-F1 elements with different numbers of BORIS binding sites depending on the length of 5’-transduced sequence. The bottom panel is the plot showing the average tag density of BORIS ChIP-Seq across the transduced sequences of different length.
10.1186/s13072-016-0084-2 Examples of BORIS binding at promoters trapped by SVA elements. The gene tracks represent cases of BORIS binding sites within genes’ promoters trapped by the indicated SVA element. The red double-headed arrows show the sequences trapped by SVAs and occupied by BORIS in K562 cells. BORIS ChIP-seq coverage and the CAGE tracks are shown for K562 cells. Expression from either minus or plus strands is shown by blue and red CAGE tracks, respectively. The particular examples are: BORIS binding site as the part of *FDX1* promoter trapped by SVA-D, *RHOT1* trapped by SVA-A, *NDUFV2*—by SVA-D, *WRD33*—SVA-F, *PHKA1* and *MMPE1*—by SVA-D.
10.1186/s13072-016-0084-2 BORIS binding sites in the promoters of unique genes captured by SVA elements.

## Abbreviations

CAGE: cap analysis of gene expression
ChIP: chromatin immunoprecipitation
ChIP-chip: microarray analysis of ChIP
ChIP-seq: NGS analysis of ChIP
CT: cancer testis (genes)
CTS: CTCF target sites
DSB: double-strand breaks
DZNep: 3-deazaneplanocin A
EMSA: electrophoretic mobility shift assay
KD: knock down
KO: knock out
NGS: next-generation sequencing
NOR: nucleolar organizer
PCA: principal component analysis
rDNA: ribosomal RNA genes
RT-qPCR: reverse transcription and quantitative polymerase chain reaction
SVA: SINE, VNTR, and Alu (transposable element)
SVD: singular value decomposition
TAD: topologically associated domains
TE: transposable elements
TR: tandem repeat
TRF: tandem repeat finder
TSSs: transcription start sites
VNTR: variable number tandem repeat
ZF: zinc finger
5-AzadCyD: 5-Aza-2′-deoxycytidine
