# Supplementary material for: The cancer-associated CTCFL/BORIS protein targets multiple classes of genomic repeats, with a distinct binding and functional preference for humanoid-specific SVA transposable elements
Source: Epigenetics Chromatin. 2016 Aug 31;9(1):35. doi: 10.1186/s13072-016-0084-2 (PMC5007689; doi:10.1186/s13072-016-0084-2)
Supplement: Supplementary file 1 — 10.1186/s13072-016-0084-2 Classes of repeats co-occupied or differentially occupied by CTCF and BORIS. [file 13072_2016_84_MOESM1_ESM.pdf]

Table S2. Classes of repeats co-occupied or differentially occupied by CTCF and BORIS.

<u>Repeats enriched for CTCF and BORIS in ChIP-chip at least 4 fold</u> (ChIP-seq ratio to input is shown)

TRF ID	Class	Type	CTCF/IN ratio	BORIS/IN ratio
117383069	LINE	L2c	305.0	17.0
117181711	LTR	MER3	22.0	9.0
117226430	LTR	LTR13	281.7	15.3
117530274	LTR	LTR12E	148.0	1976.0
117530440	LTR	LTR12E	105.3	1395.0
117530489	LTR	LTR12E	77.5	1014.0
117530558	LTR	LTR12E	97.0	1294.0
117583547	LTR	LTR13	554.0	25.0
117736110	LTR	MLT2A1	62.0	6.0
116978074	satellite	TAR1	386.9	38.2
116978075	satellite	TAR1	364.8	61.0
117028136	satellite	TAR1	530.7	118.2
117028197	satellite	TAR1	306.8	34.7
117059113	satellite	TAR1	419.3	1505.0
117059114	satellite	TAR1	419.3	1505.0
117091843	satellite	SST1	50.5	205.2
117139191	satellite	TAR1	511.1	290.1
117139192	satellite	TAR1	511.1	290.1
117152426	satellite	TAR1	530.7	118.2
117152434	satellite	TAR1	420.9	259.0
117152435	satellite	TAR1	542.9	331.4
117287537	satellite	TAR1	424.1	96.0
117287538	satellite	TAR1	500.9	174.5
117373295	satellite	TAR1	127.7	26.5
117373302	satellite	TAR1	406.9	136.5
117412055	satellite	TAR1	435.7	1541.0
117412056	satellite	TAR1	435.7	1541.0
117412206	satellite	TAR1	313.8	35.0
117412207	satellite	TAR1	324.6	36.1
117457878	satellite	TAR1	523.1	313.3
117618860	satellite	TAR1	264.8	27.2
117729434	satellite	TAR1	242.3	41.3
117764023	satellite	TAR1	335.6	192.8
117764024	satellite	TAR1	375.9	202.6
117764034	satellite	TAR1	211.5	40.4
117764035	satellite	TAR1	125.5	19.5
117802978	satellite	TAR1	425.4	244.7
117802979	satellite	TAR1	506.2	293.4
117802980	satellite	TAR1	344.2	202.3
116929700	simple short	(CAG)n	292.0	24.8
116929701	simple short	(CAG)n	167.0	17.3
116986658	simple short	(CAGCG)n	117.0	12.0
117406683	simple short	(CTG)n	15.7	5.7
116930440	SIMPLE TR	NA	13.3	12.4
117028615	SIMPLE TR	NA	181.8	245.2
117028616	SIMPLE TR	NA	39.0	18.0
117037259	SIMPLE TR	NA	116.0	9.0
117037260	SIMPLE TR	NA	108.0	13.0
117082685	SIMPLE TR	NA	25.0	56.0
117101237	SIMPLE TR	NA	48.0	15.0

117114743	SIMPLE TR	NA	180.4	16.6
117114744	SIMPLE TR	NA	134.2	11.4
117114745	SIMPLE TR	NA	116.3	9.9
117114746	SIMPLE TR	NA	213.5	18.3
117114747	SIMPLE TR	NA	125.4	11.0
117114748	SIMPLE TR	NA	146.0	8.0
117114749	SIMPLE TR	NA	105.0	8.3
117152436	SIMPLE TR	NA	358.1	227.5
117167017	SIMPLE TR	NA	403.7	34.3
117167018	SIMPLE TR	NA	411.5	34.7
117167056	SIMPLE TR	NA	251.0	22.0
117167057	SIMPLE TR	NA	182.0	17.0
117167058	SIMPLE TR	NA	173.0	17.0
117167059	SIMPLE TR	NA	102.0	9.0
117167062	SIMPLE TR	NA	175.8	12.6
117167063	SIMPLE TR	NA	174.8	12.6
117167064	SIMPLE TR	NA	120.5	8.5
117167065	SIMPLE TR	NA	107.2	9.0
117167066	SIMPLE TR	NA	81.2	6.0
117167067	SIMPLE TR	NA	151.8	11.8
117167531	SIMPLE TR	NA	319.3	29.7
117167532	SIMPLE TR	NA	342.3	33.7
117167533	SIMPLE TR	NA	480.0	40.0
117167566	SIMPLE TR	NA	282.0	22.0
117167569	SIMPLE TR	NA	214.0	17.0
117167572	SIMPLE TR	NA	80.0	5.7
117167573	SIMPLE TR	NA	80.0	5.9
117167574	SIMPLE TR	NA	132.0	10.8
117167575	SIMPLE TR	NA	99.2	8.9
117167913	SIMPLE TR	NA	304.0	26.0
117167914	SIMPLE TR	NA	244.0	22.0
117167928	SIMPLE TR	NA	92.8	7.5
117167929	SIMPLE TR	NA	221.0	15.0
117167930	SIMPLE TR	NA	131.0	15.0
117167967	SIMPLE TR	NA	397.0	32.7
117167968	SIMPLE TR	NA	685.0	69.0
117167969	SIMPLE TR	NA	401.0	33.0
117181712	SIMPLE TR	NA	23.0	9.0
117213978	SIMPLE TR	NA	115.8	11.2
117213979	SIMPLE TR	NA	102.1	10.0
117213980	SIMPLE TR	NA	385.0	40.0
117213981	SIMPLE TR	NA	211.1	18.5
117213982	SIMPLE TR	NA	221.6	18.6
117213983	SIMPLE TR	NA	197.0	19.2
117213984	SIMPLE TR	NA	120.6	12.0
117213985	SIMPLE TR	NA	280.0	31.0
117213986	SIMPLE TR	NA	282.7	31.7
117213987	SIMPLE TR	NA	32.0	7.0
117213989	SIMPLE TR	NA	80.6	8.4
117213990	SIMPLE TR	NA	88.1	9.3
117214003	SIMPLE TR	NA	413.0	40.0
117214016	SIMPLE TR	NA	85.7	7.0
117214017	SIMPLE TR	NA	98.5	6.0
117214020	SIMPLE TR	NA	99.0	6.0
117214021	SIMPLE TR	NA	87.3	5.7

117214023	SIMPLE TR	NA	119.7	6.3
117214024	SIMPLE TR	NA	119.3	6.3
117214025	SIMPLE TR	NA	174.0	11.0
117214040	SIMPLE TR	NA	374.1	32.0
117214041	SIMPLE TR	NA	295.7	25.2
117214076	SIMPLE TR	NA	211.0	24.0
117214077	SIMPLE TR	NA	351.0	35.0
117214078	SIMPLE TR	NA	316.0	28.3
117214079	SIMPLE TR	NA	424.5	38.5
117214097	SIMPLE TR	NA	463.5	43.5
117214098	SIMPLE TR	NA	133.0	18.0
117214122	SIMPLE TR	NA	233.0	20.0
117214144	SIMPLE TR	NA	415.8	30.5
117214145	SIMPLE TR	NA	556.3	44.0
117214146	SIMPLE TR	NA	832.0	66.0
117214158	SIMPLE TR	NA	292.3	32.3
117214159	SIMPLE TR	NA	328.0	38.0
117214163	SIMPLE TR	NA	211.0	19.0
117214167	SIMPLE TR	NA	32.0	6.0
117214168	SIMPLE TR	NA	215.8	24.0
117214169	SIMPLE TR	NA	287.0	35.7
117214170	SIMPLE TR	NA	147.0	10.0
117214172	SIMPLE TR	NA	256.0	24.0
117214203	SIMPLE TR	NA	551.0	56.0
117214204	SIMPLE TR	NA	555.0	58.0
117214205	SIMPLE TR	NA	452.0	33.0
117214207	SIMPLE TR	NA	214.0	15.0
117214214	SIMPLE TR	NA	224.0	33.0
117214216	SIMPLE TR	NA	372.0	41.0
117214217	SIMPLE TR	NA	123.0	19.0
117243774	SIMPLE TR	NA	333.3	1880.0
117305874	SIMPLE TR	NA	37.0	435.0
117373567	SIMPLE TR	NA	283.3	15.0
117409234	SIMPLE TR	NA	16.3	40.3
117412080	SIMPLE TR	NA	25.0	6.0
117412159	SIMPLE TR	NA	116.2	319.8
117507932	SIMPLE TR	NA	167.3	1165.0
117507938	SIMPLE TR	NA	124.0	849.0
117507941	SIMPLE TR	NA	342.5	2349.0
117507950	SIMPLE TR	NA	344.5	2350.0
117507956	SIMPLE TR	NA	335.0	2278.0
117508779	SIMPLE TR	NA	130.4	355.0
117530210	SIMPLE TR	NA	18.0	17.0
117530441	SIMPLE TR	NA	18.0	17.0
117530488	SIMPLE TR	NA	19.5	19.5
117530559	SIMPLE TR	NA	18.0	17.0
117530825	SIMPLE TR	NA	18.0	17.0
117562409	SIMPLE TR	NA	110.9	304.9
117562410	SIMPLE TR	NA	108.8	303.1
117562411	SIMPLE TR	NA	112.5	321.7
117701167	SIMPLE TR	NA	40.0	1047.0
117722158	SIMPLE TR	NA	95.0	25.5
117724864	SIMPLE TR	NA	14.0	31.0
117738027	SIMPLE TR	NA	96.0	19.0
117758464	SIMPLE TR	NA	69.0	9.0

117758465	SIMPLE TR	NA	60.0	9.0
117764133	SIMPLE TR	NA	41.4	138.6
117768990	SIMPLE TR	NA	12.0	36.0
117803090	SIMPLE TR	NA	110.9	304.9
117803091	SIMPLE TR	NA	108.8	303.1
117803092	SIMPLE TR	NA	112.5	321.7
117803165	SIMPLE TR	NA	121.1	331.7
117839149	SIMPLE TR	NA	50.0	218.0
117839201	SIMPLE TR	NA	98.0	581.0
117875281	SIMPLE TR	NA	429.0	513.0
117875282	SIMPLE TR	NA	150.0	188.3

<u>Repeats enriched for CTCF in ChIP-chip at least 4 fold, with no enrichment for BORIS</u>
(ChIP-seq ratio is shown)

TRF ID	Class	Type	CTCF/BORIS ratio
117868503	Satellite	ALR/Alpha	4.6
117228333	Satellite	ALR/Alpha	4.3
117061439	Satellite	ALR/Alpha	4.0
117325607	SINE	AluJb	5.6
117469204	SINE	AluJr	5.2
117229411	SINE	AluSc	5.3
117750246	SINE	AluSc8	5.4
116936795	SINE	AluSg	5.5
117303127	SINE	AluSg	5.3
117059756	SINE	AluSq	5.1
117124943	SINE	AluSq2	5.6
117765410	SINE	AluSx	5.3
117806747	SINE	AluSx	5.3
117004336	SINE	AluSx	5.0
117620091	SINE	AluSx1	5.4
116970147	SINE	AluSx3	5.5
117513433	SINE	AluSx3	4.8
117171338	SINE	AluSz	5.6
116991347	SINE	AluSz	5.5
117808800	SINE	AluSz	5.4
117372302	SINE	AluY	5.4
116996579	SINE	AluY	5.0
117004337	SINE	AluY	5.0
117613361	SINE	AluYa5	4.8
117631115	SINE	AluYc	4.9
117232871	SINE	FLAM_C	5.8
117362109	LINE	L1PA12	8.5
117599577	LTR	LTR12	6.8
117063031	LTR	LTR12 ERV1	4.9
117594511	LTR	LTR12C	6.4
117650263	LTR	LTR12D	7.8
117650264	LTR	LTR12D	4.6
117190211	LTR	LTR12F	6.0
117862943	LTR	LTR7B	8.9
117849194	LTR	MER11B	5.3
117222182	SIMPLE TR	NA	10.6
117365770	SIMPLE TR	NA	5.6
117329295	SIMPLE TR	NA	3.8

Repeats enriched for BORIS in ChIP-chip at least 4 fold, with no enrichment for CTCF
(ChIP-seq ratio is shown)

TRF ID	Class	Type	BORIS/CTCF ratio
117100136	Other	SVA_A	2.4
117630598	Other	SVA_B	2.9
117691562	Other	SVA_D	12.4
117077974	Other	SVA_D	2.7
117711345	Other	SVA_D	2.7
117254541	Other	SVA_D	1.8
117422627	Other	SVA_E	11.6
117329211	Other	SVA_E	2.4
117633823	Other	SVA_F	11.1
117105907	Other	SVA_F	1.8
