# Supplementary figures and images for: The cancer-associated CTCFL/BORIS protein targets multiple classes of genomic repeats, with a distinct binding and functional preference for humanoid-specific SVA transposable elements
Source: Epigenetics Chromatin. 2016 Aug 31;9(1):35. doi: 10.1186/s13072-016-0084-2 (PMC5007689; doi:10.1186/s13072-016-0084-2)

**A**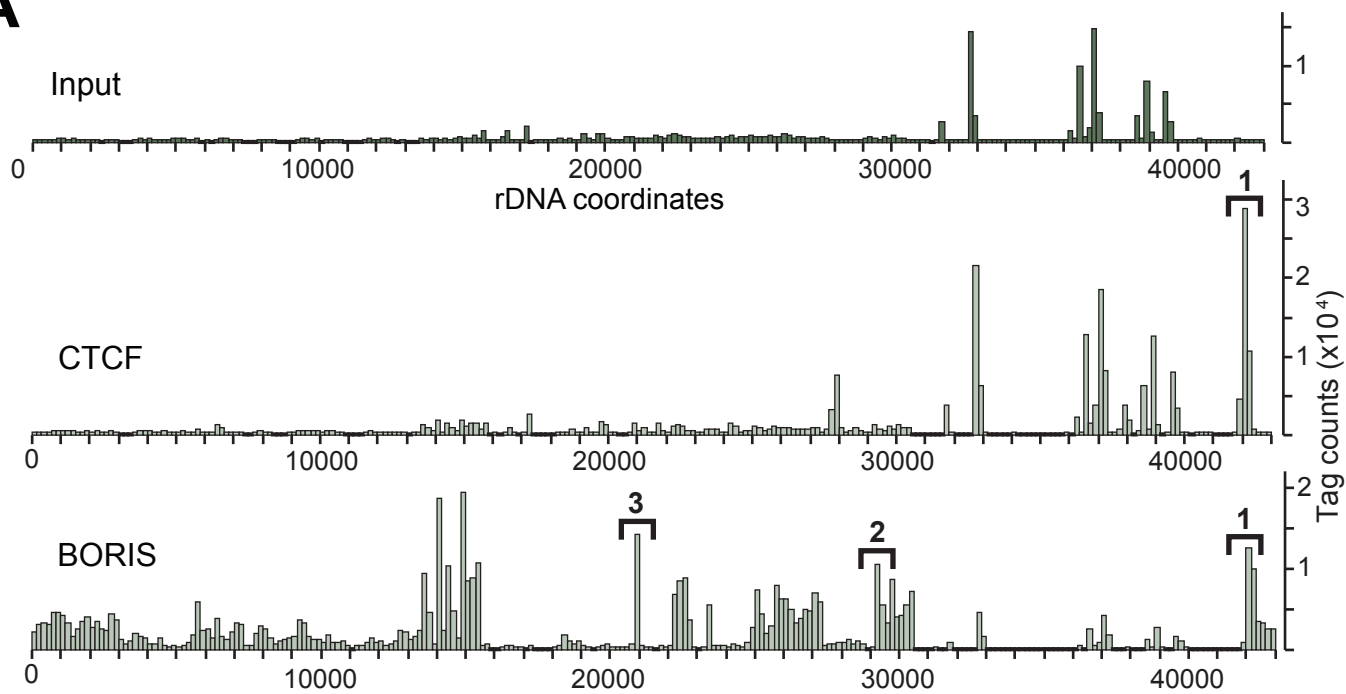**B**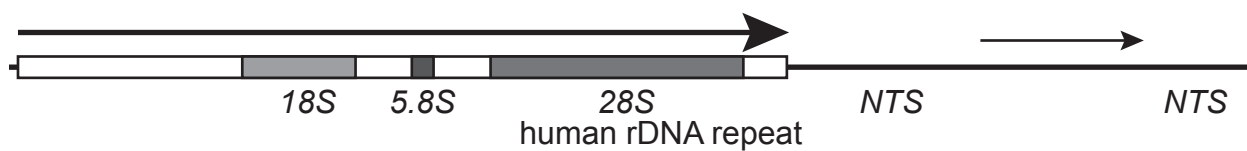**C**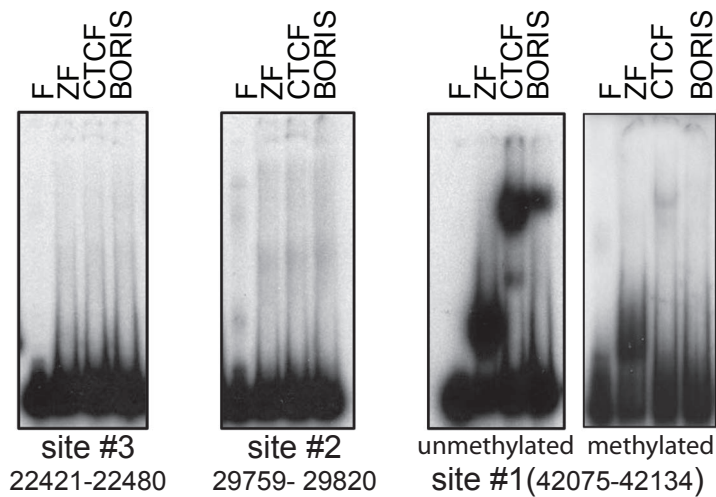**D**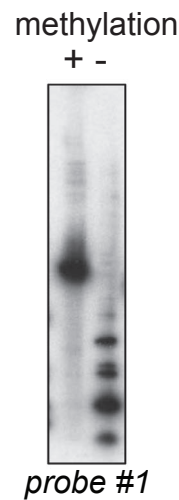

Supplement: Supplementary file 2 — 10.1186/s13072-016-0084-2 BORIS binds at the same regulatory site as CTCF in rDNA. (A) The distribution of Chip-seq tags across the consensus rDNA repeat is shown for the input, CTCF ChIP-seq and BORIS ChIP-seq. The sites chosen for EMSA are indicated with brackets. (B) The corresponding structure of “canonical” rDNA repeat. The long arrow corresponds to the Pol I transcript; the short arrow—noncoding RNA; NTS—non-transcribed spacers. (C) EMSA of the chosen rDNA sites confirming that the known CTCF site in PolI promoter is co-occupied by CTCF in BORIS, while sampling of BORIS-only putative sites shows that there is no BORIS binding to these sites in vitro. (D) The assessment of CpG methylation for probe #3 used in (C) using the diagnostic digestion with Aci I endonuclease, before and after methylation. [file 13072_2016_84_MOESM2_ESM.pdf]

**A**

Delta-47  
SVA-D\_Repbse

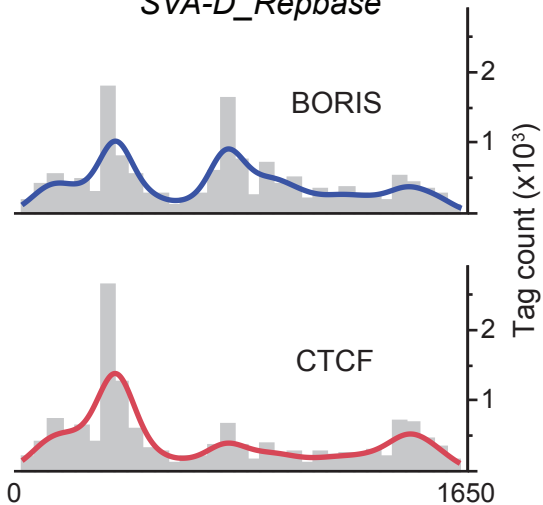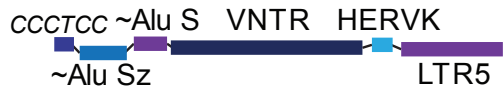**B**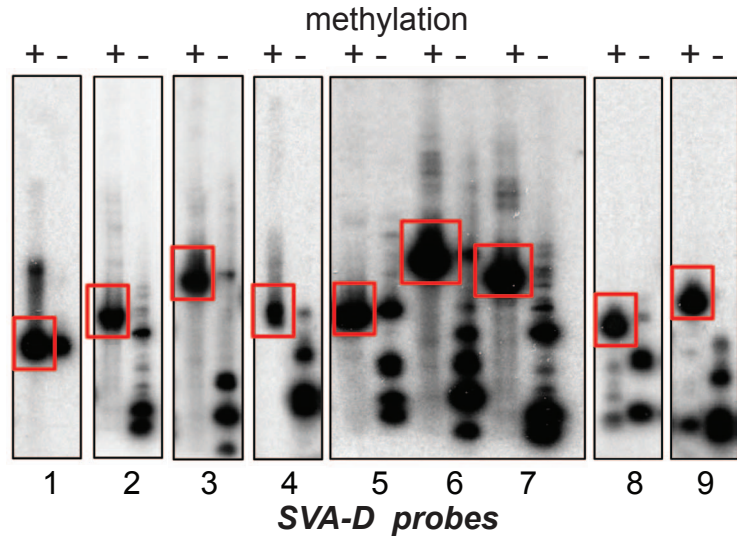

Supplement: Supplementary file 4 — 10.1186/s13072-016-0084-2 Extended analyses of SVA-D. (A) BORIS binding peaks at SVA VNTRs in Delta-47 cells. The ChIP-seq tag density distribution for the full-length SVA-D element from Repbase indicates that BORIS retains preference for VNTR region event in this cell line completely unrelated to K562 and with a substantially lower BORIS expression level. The normalized counts were binned along the DNA sequence (histogram bars) with the smoothing line added. (B) The assessment if CpG methylation of oligonucleotides used in EMSA. DNA fragments were digested by the methylation-sensitive endonuclease Aci I before and after methylation. [file 13072_2016_84_MOESM4_ESM.pdf]

# 75 SVA expressed in control

**A**

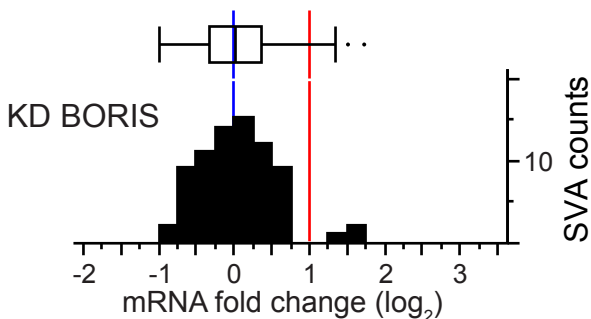

**B**

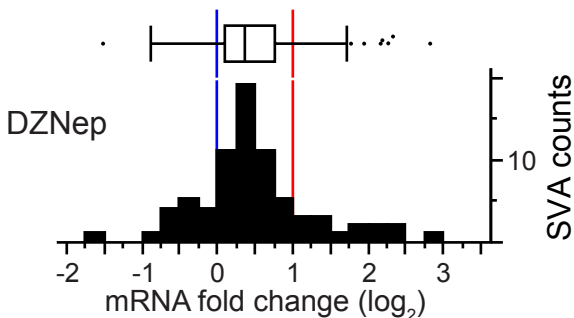

**C**

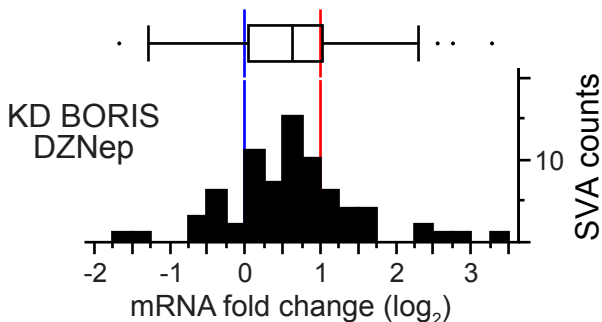

Supplement: Supplementary file 5 — 10.1186/s13072-016-0084-2 The expression of SVA elements that are transcribed in K562 cells is not affected by BORIS dosage. RNA-seq differential ratio distribution for 75 SVA elements, which were apparently transcriptionally active in the untreated K562 cells (i.e., over 10 normalized counts in empty vector control). Only elements longer than 1 Kb were included in analysis. Shown are the graphs for BORIS KD K562 cells, K562 treated with DZNep and the combination of both treatments. [file 13072_2016_84_MOESM5_ESM.pdf]

**A**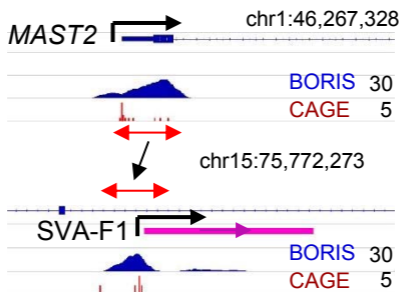**B**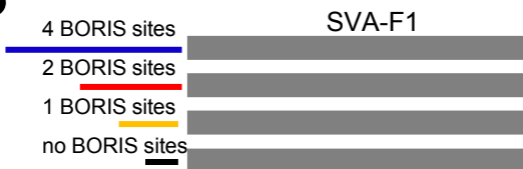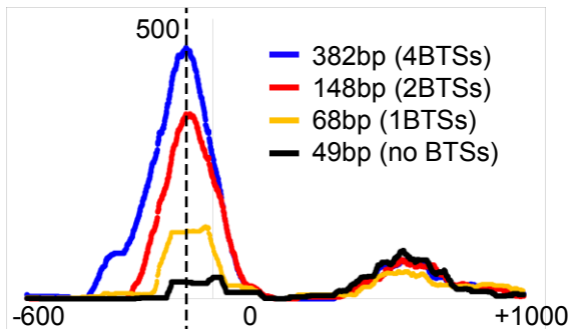

Supplement: Supplementary file 6 — 10.1186/s13072-016-0084-2 SVA elements capture BORIS binding sites from unique gene promoters. (A) BORIS ChIP-seq and deep CAGE (Cap Analysis of Gene Expression)-seq (ENCODE data) coverage tracks for the MAST2 gene (upper track) and for the SVA-F1 element (lower track) in K562 cells. BORIS occupancy at the MAST2 first exon sequence coincided with the multiple transcription start sites (TSS) for MAST2 and SVA-F1 family expression in K562 cells. The black arrows show the direction of transcription based on CAGEs enrichment on plus strand. The red double-headed arrows show the MAST2 sequence captured by SVA-F1 family from the MAST2 gene. (B) ChIP-seq enrichment of BORIS occupancy depends on the number of BORIS binding sites in the transduced sequences. The top panel is the schematic representation of SVA-F1 elements with different numbers of BORIS binding sites depending on the length of 5’-transduced sequence. The bottom panel is the plot showing the average tag density of BORIS ChIP-Seq across the transduced sequences of different length. [file 13072_2016_84_MOESM6_ESM.pdf]

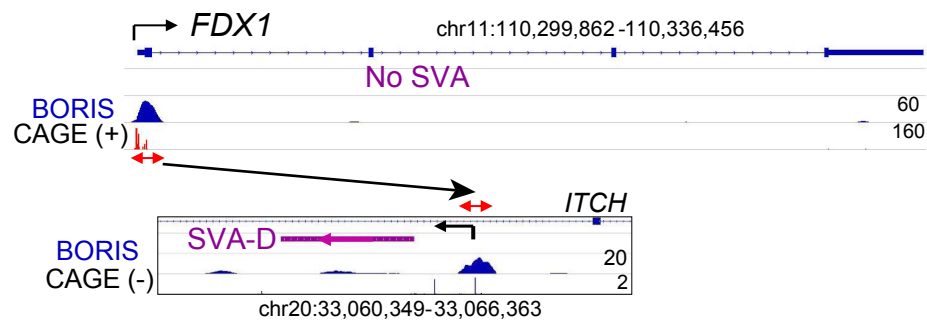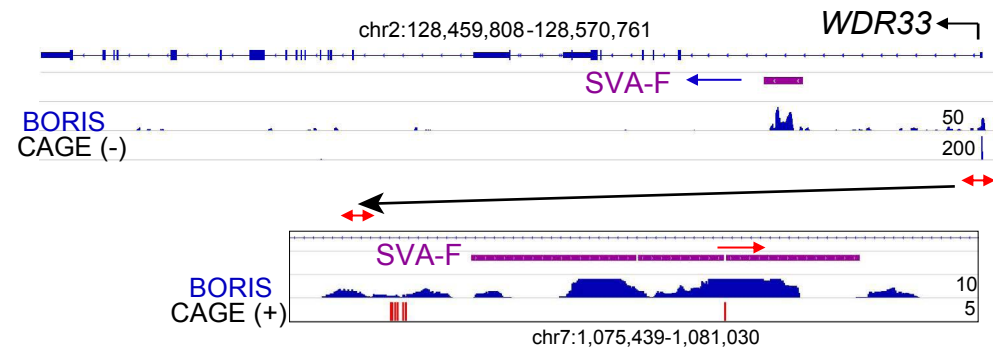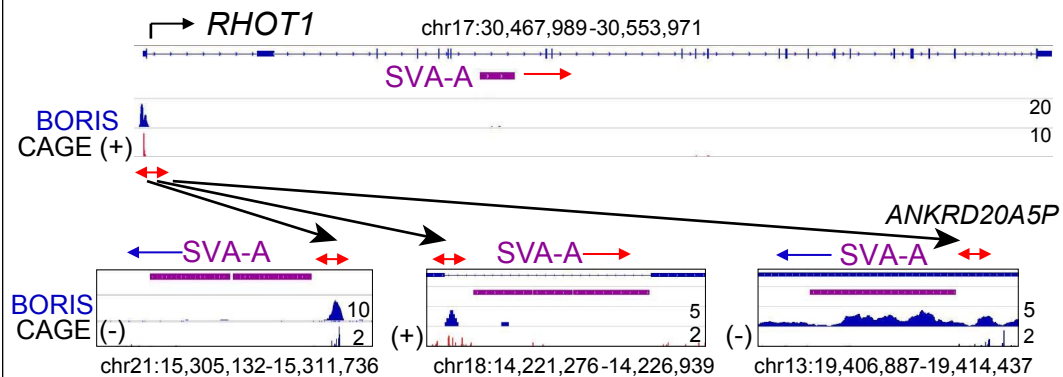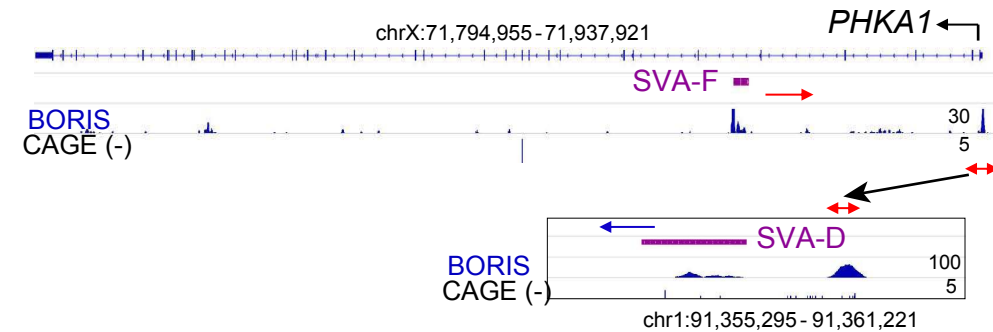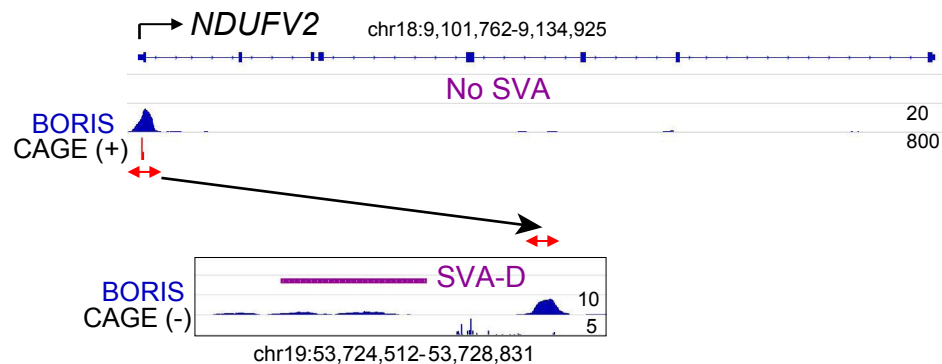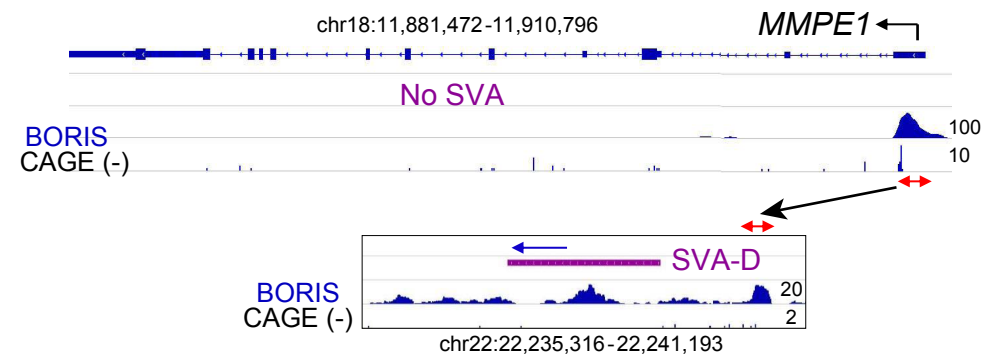

Supplement: Supplementary file 7 — 10.1186/s13072-016-0084-2 Examples of BORIS binding at promoters trapped by SVA elements. The gene tracks represent cases of BORIS binding sites within genes’ promoters trapped by the indicated SVA element. The red double-headed arrows show the sequences trapped by SVAs and occupied by BORIS in K562 cells. BORIS ChIP-seq coverage and the CAGE tracks are shown for K562 cells. Expression from either minus or plus strands is shown by blue and red CAGE tracks, respectively. The particular examples are: BORIS binding site as the part of FDX1 promoter trapped by SVA-D, RHOT1 trapped by SVA-A, NDUFV2—by SVA-D, WRD33—SVA-F, PHKA1 and MMPE1—by SVA-D. [file 13072_2016_84_MOESM7_ESM.pdf]
