# Supplementary material for: The cancer-associated CTCFL/BORIS protein targets multiple classes of genomic repeats, with a distinct binding and functional preference for humanoid-specific SVA transposable elements
Source: Epigenetics Chromatin. 2016 Aug 31;9(1):35. doi: 10.1186/s13072-016-0084-2 (PMC5007689; doi:10.1186/s13072-016-0084-2)
Supplement: Supplementary file 3 — 10.1186/s13072-016-0084-2 EMSA oligonucleotides. [file 13072_2016_84_MOESM3_ESM.pdf]

Table S1. EMSA oligonucleotides

**Primers to amplify DNA fragments overlapping SVA- D repeat for EMSA**	
CCCTCC- repeat (EMSA_Probe1)	5´CCATTCTCAGGCAGGCTTTCTTCCCAC
	5´CAGGCAGGGAGGTTGCAGTGAGCCGAGATGG
AluSz (EMSA_Probe2)	5´CGAGCCGAAGCTGGACTGTACTGCTGCCATCTC
	5´GAACCAGACTCCGTCTGCAATCCCGGCACCTCGG
AluS (EMSA_Probe3)	5´CACCAGCCTCGGCCTCCCGAGGTGCCGGGATTG
	5´CAGAGGGGCTCCTCACGTCCCAGACGATGGGTGGC
HERVK (EMSA_Probe7)	5´CTCTGCCCGGCCAGCCGCCCAGTCCGGGAGGGAG
	5´CAATCTTTTCCCCACCTTTCCCCTCCTTCTATTC
HERVK (EMSA_Probe8)	5´GCTCATTGAGAGCGGGCCATGATGACAATGGCG
	5´CCATTTAACCCTGAGTGGACACAGCACATGTTTCAG
LTR5 (EMSA_Probe9)	5´CAACCCTGTGCTCTCTGAAACATGTGCTGTGTC
	5´GCAGTGGGGGATTTGGCAGGGTCATAGGACAATAGTG
**Synthesized oligonucleotide probes for EMSA overlapping SVA- D repeat**	
VNTR (EMSA_Probe4)	5´GCCACCCATCGTCTGGGACGTGAGGAGCCCCTCTGCCTGGCTGCCCAG-TCTGGAAAGTGAGGAGCGCTTCTTCCCGGCCGCCATCCCATCTAGGAA-GTGAGGAGTGCCTCTTCCCGGCCACCATCCCATCTAGGAAGTGAGGAG-CGTCTCTGCCCGCCGCCCATTGTCTGAGATGTGGGGAGCGCC
VNTR (EMSA_Probe5)	5´TCTGCCCGCCGCCCATTGTCTGAGATGTGGGGAGCGCCTCTGCCCCGC-CGCCCCGTCTGGGATGTGAGGAGCGCCTCTGCCCGGCCGCGACCCCGTCTG-GGAGGTGAGGAGACCCTCCGCCCGGCAACCGCCCCGTCTGAGAAGTGA-GGAGCCCCTCCGCCCGGCAGCCGCCCCGTCTGAGAAGTGAAGAGCCCCTCC-GCCCGGCAGCCACCCCGTTTGGGAGGTGAGGAGCGTCTCCGCCCTGCAGCC-ACCCCGTTCGGGAGGGAGGTGGGGGGGTCAGCCCCCCCGCCGGCCGGCCG-CCC
VNTR (EMSA_Probe6)	5´GGGGGGTCAGCCCCCCCGCCGGCCGGCCGCCCAGTCCGGGAGGGAGG-TGGGGTGTCAGCCCCCCGCCCGGCCAGCCGCCCCGTCCGGGAGGTGAGGGG-CGCCTCTGCCCGGCCGCCCCTACTGGGAGGTGAGGAGCCCCTCTGCCCGGC-CAGCCGCCCAGTCCGGGAGGGAGGTGG
rDNA probe 3 (22421-22480, putative BORIS only site)	5’CTCTCTCTCTCTGTCTGTCTGTCTGTCTCTCTCTCTCTCTCTGTGC-CTATCTTCTGTC
rDNA probe 2 (29759- 29820, putative BORIS only site)	5’CTCTCTCTCTCTCTCTCTCTCTCTGTCTTTGTCTTTCTTTCTGTCTCT-GTCTCTCTCTCTCT
rDNA probe 1 (42075-42134, both CTCF and BORIS bind)	5’TGACGTCCGCGGCGGTTGTCGGGCTCCATCTGGCGGCCGCTTTGAGAT-CGTGCTCTCGGC
