# Supplementary material for: The cancer-associated CTCFL/BORIS protein targets multiple classes of genomic repeats, with a distinct binding and functional preference for humanoid-specific SVA transposable elements
Source: Epigenetics Chromatin. 2016 Aug 31;9(1):35. doi: 10.1186/s13072-016-0084-2 (PMC5007689; doi:10.1186/s13072-016-0084-2)
Supplement: Supplementary file 8 — 10.1186/s13072-016-0084-2 BORIS binding sites in the promoters of unique genes captured by SVA elements. [file 13072_2016_84_MOESM8_ESM.pdf]

Table S3. BORIS binding sites in the promoters of unique genes captured by SVA elements.

BORIS binding in promoter		BORIS binding captured by SVA	SVA repeats with captured BORIS binding site		
position	gene	position	position	SVA type	strand
chr17:30,469,223-30,469,744	RHOT1	chr21:15,309,614-15,309,849	chr21:15,306,573-15,309,317	SVA-A	Minus
chr17:30,469,223-30,469,744	RHOT1	chr18:14,221,817-14,222,039	chr18:14,222,333-14,225,661	SVA-A	Plus
chr17:30,469,223-30,469,744	RHOT1	chr13:19412205-19412362	chr13:19,409,920-19,411,923	SVA-A	Minus
chr18:9,102,532-9,102,970	NDUFV2	chr19:53,727,918-53,728,132	chr19:53,725,481-53,726,903	SVA-D	Plus
chr11:110,300,583-110,301,206	FDX1	chr20:33,064,095-33,064,297	chr20:33,061,965-33,063,512	SVA-D	Minus
chr2:128,568,591-128,568,975	WDR33	chr7:1,076,326-1,076,465	chr7:1,076,932-1,079,836	SVA-F	Plus
chrX:71,933,796-71,934,373	PHKA1	chr1:91,359,237-91,359,566	chr1:91,356,737-91,358,141	SVA-D	Minus
chr18:11,908,176-11,908,985	MPPE1	chr22:22,240,215-22,240,328	chr22:22,237,234-22,239,125	SVA-D	Minus
chr10:43,932,055-43,932,807	ZNF487	chr7:77,137,691-77,137,828	chr7:77,137,850-77,139,906	SVA-F	Plus
chr10:43,932,055-43,932,807	ZNF488	chr2:178,809,388-178,809,493	chr2:178,809,585-178,812,513	SVA-F	Plus
chr16:71,757,398-71,758,231	PHLPP2	chr16:48,093,998-48,094,109	chr16:48,092,433-48,093,586	SVA-A	Minus
chr17:17,875,403-17,875,925	TOM1L2	chr3:51,891,479-51,891,585	chr3:51,891,964-51,893,692	SVA-D	Plus
chr7:12,726,284-12,726,673	ARL4A	chr4:40,787,905-40,788,042	chr4:40,789,194-40,789,354	SVA-A	Plus
